# Supplementary material for: A sensitive and affordable multiplex RT-qPCR assay for SARS-CoV-2 detection
Source: PLoS Biol. 2020 Dec 15;18(12):e3001030. doi: 10.1371/journal.pbio.3001030 (PMC7771873; doi:10.1371/journal.pbio.3001030)
Supplement: S1 Table — Values used for Fig 2A and S1A Fig. Cq, cycle quantification; SARS-CoV-2, Severe Acute Respiratory Syndrome Coronavirus 2. (PDF) [file pbio.3001030.s001.pdf]

S1 Table. N1E-RP and N2E-RP assay Cq values for SARS-CoV-2 RNA controls (1 to 10,000 copies) pre- and post-extraction.

# copies	10,000	1,000	100	50	10	1
**N1, N1E-RP assay pre-extraction**						
Rep 1	22.83	26.49	30.30	31.42	33.49	35.87
Rep 2	23.36	26.62	30.50	31.05	34.43	Undetermined
Rep 3	23.30	26.77	29.32	32.38	34.94	Undetermined
Mean	23.17	26.62	30.04	31.62	34.29	35.87
**E gene, N1E-RP pre-extraction**						
Rep 1	24.26	27.98	31.37	33.60	36.26	Undetermined
Rep 2	24.74	27.73	32.73	35.22	Undetermined	Undetermined
Rep 3	24.43	27.90	31.34	32.36	Undetermined	Undetermined
Mean	24.48	27.87	31.81	33.73	36.26	Undetermined
**N2, N2E-RP assay pre-extraction**						
Rep 1	26.63	30.49	33.99	34.63	37.34	38.72
Rep 2	26.88	29.99	34.01	35.06	Undetermined	Undetermined
Rep 3	26.70	30.58	33.79	37.34	36.99	Undetermined
Mean	26.74	30.35	33.93	34.47	37.16	38.72
**E gene, N2E-RP assay pre-extraction**						
Rep 1	25.50	28.71	32.84	33.37	35.25	Undetermined
Rep 2	25.70	28.71	33.05	33.24	35.93	36.64
Rep 3	25.50	29.56	32.29	33.85	35.05	Undetermined
Mean	25.57	28.99	32.73	33.49	35.41	36.64
**N1, N1E-RP assay post-extraction**						
Rep 1	25.64	29.02	33.14	33.59	35.09	Undetermined
Rep 2	25.14	28.80	33.41	33.58	35.37	36.14
Rep 3	25.50	29.01	33.22	33.87	37.62	37.04
Mean	25.43	28.94	33.25	33.68	36.03	36.59
**E gene, N1E-RP assay post-extraction**						
Rep 1	25.99	29.24	33.26	35.01	35.59	Undetermined
Rep 2	25.39	28.88	34.04	33.87	33.64	Undetermined
Rep 3	25.61	29.22	32.47	33.81	Undetermined	Undetermined
Mean	25.66	29.11	33.26	34.23	34.61	Undetermined
**N2, N2E-RP assay post-extraction**						
Rep 1	28.69	32.39	36.86	36.01	38.35	Undetermined
Rep 2	28.33	31.69	35.75	35.57	38.17	Undetermined
Rep 3	28.79	32.56	35.40	36.21	Undetermined	Undetermined
Mean	28.60	32.21	36.01	35.93	38.26	Undetermined
**E gene, N2E-RP assay post-extraction**						
Rep 1	26.72	29.89	33.85	33.40	Undetermined	Undetermined
Rep 2	26.45	30.01	33.38	34.53	35.87	Undetermined
Rep 3	26.96	30.30	33.79	34.56	36.10	36.40
Mean	26.71	30.07	33.67	34.16	35.98	36.40

Values used for Fig 2A and S1A Fig.
