# Supplementary material for: A sensitive and affordable multiplex RT-qPCR assay for SARS-CoV-2 detection
Source: PLoS Biol. 2020 Dec 15;18(12):e3001030. doi: 10.1371/journal.pbio.3001030 (PMC7771873; doi:10.1371/journal.pbio.3001030)
Supplement: S2 Table — Values used for Fig 2B and S1B Fig. Cq, cycle quantification; SARS-CoV-2, Severe Acute Respiratory Syndrome Coronavirus 2. (PDF) [file pbio.3001030.s002.pdf]

S2 Table. N1E-RP and N2E-RP assay Cq values for cultured SARS-CoV-2 dilution series (before and after re-extraction.

	neat	10^{-1}	10^{-2}	10^{-3}	10^{-4}	10^{-5}	10^{-6}
**N1, N1E-RP assay**							
1	22.53	26.46	29.42	32.21	37.19	UD	UD
2	22.83	26.03	29.87	32.79	UD	UD	UD
3	22.40	25.96	29.35	33.48	37.16	UD	UD
Mean	22.59	26.15	29.55	32.83	37.18	UD	UD
**E gene, N1E-RP assay**							
1	21.31	24.77	27.97	31.35	34.22	UD	UD
2	21.38	24.38	28.03	31.50	35.27	UD	UD
3	21.35	24.41	27.84	31.55	35.63	UD	UD
Mean	21.35	24.52	27.94	31.46	35.04	UD	UD
**N2, N2E-RP assay**							
1	23.08	26.76	29.95	33.56	37.21	38.06	UD
2	23.36	26.64	29.79	34.81	36.50	38.29	UD
3	23.32	26.46	30.14	33.49	36.44	UD	UD
Mean	23.25	26.62	29.96	33.95	36.72	38.17	UD
**E gene, N2E-RP assay**							
1	22.00	25.48	28.78	32.41	36.34	UD	UD
2	22.22	25.42	28.57	32.59	UD	40.63	UD
3	22.15	25.31	28.87	32.31	39.68	UD	UD
Mean	22.12	25.40	28.74	32.44	38.01	40.63	UD
	**0.5**	10^{-1}	10^{-2}	10^{-3}	10^{-4}	10^{-5}	10^{-6}
**N1, N1E-RP assay after re-extraction**							
1	24.95	27.48	30.79	34.27	UD	UD	UD
2	24.93	27.54	31.08	34.77	37.44	UD	UD
3	25.04	27.12	30.69	34.26	UD	UD	UD
Mean	24.97	27.38	30.85	34.44	37.44	UD	UD
**E gene, N1E-RP assay after re-extraction**							
1	23.85	26.56	29.63	33.49	UD	UD	UD
2	23.86	26.53	29.85	33.30	UD	UD	UD
3	24.18	26.59	29.59	32.77	35.54	UD	UD
Mean	23.96	26.56	29.69	33.19	35.54	UD	UD
**N2, N2E-RP assay after re-extraction**							
1	25.90	28.27	31.84	35.46	37.68	UD	UD
2	25.76	28.36	31.78	34.97	UD	UD	UD
3	25.53	28.27	31.13	34.36	UD	UD	UD
Mean	25.73	28.30	31.58	34.93	37.68	UD	UD
**E gene, N2E-RP assay after re-extraction**							
1	24.86	27.37	30.73	33.22	UD	UD	UD
2	25.00	27.55	30.81	34.54	UD	UD	UD
3	23.65	27.62	31.25	34.19	35.96	UD	UD
Mean	24.51	27.51	30.93	33.99	35.96	UD	UD

Values used for Fig 2B and S1B Fig.
