# Supplementary material for: A sensitive and affordable multiplex RT-qPCR assay for SARS-CoV-2 detection
Source: PLoS Biol. 2020 Dec 15;18(12):e3001030. doi: 10.1371/journal.pbio.3001030 (PMC7771873; doi:10.1371/journal.pbio.3001030)
Supplement: S3 Table — Values used for Fig 2C. Cq, cycle quantification; QCMD, Quality Control for Molecular Diagnostics; SARS-CoV-2, Severe Acute Respiratory Syndrome Coronavirus 2. (PDF) [file pbio.3001030.s003.pdf]

S3 Table. N1E-RP, N2E-RP and TaqPath assays all correctly identify SARS-CoV-2 positive QCMD quality control samples

			N1E-RP assay			N2E-RP assay			TaqPath assay			Conclusion		
QCMD Sample	Content [a]	Log10 dPCR Copies/ml [b]	N1	E	PhHV	N2	E	PhHV	N	ORF1ab	S	N1E-RP	N2E-RP	Taq Path
CVOP20S2-01	SARS-CoV-2	4.30	29.72	28.98	32.22	29.58	29.14	32.98	28.06	28.21	28.15	P	P	P
CVOP20S2-02	Coronavirus – NL63	4.64	UD	UD	33.54	UD	UD	32.79	UD	UD	UD	N	N	N
CVOP20S2-03	SARS-CoV-2	3.30	32.26	31.62	32.66	32.70	31.8	32.52	30.24	29.22	30.02	P	P	P
CVOP20S2-04	Coronavirus – OC43	4.03	UD	UD	32.64	UD	UD	33.01	UD	UD	UD	N	N	N
CVOP20S2-05	Transport medium	-	UD	UD	32.54	UD	UD	32.45	UD	UD	UD	N	N	N
CVOP20S2-06	SARS-CoV-2	4.30	29.77	28.94	32.90	29.56	28.82	32.67	27.96	27.79	27.63	P	P	P
CVOP20S2-07	SARS-CoV-2	5.30	26.18	25.42	31.64	26.20	25.44	31.93	24.63	24.52	24.65	P	P	P
CVOP20S2-08	SARS-CoV-2	2.30	35.69	34.55	33.02	35.66	35.88	32.74	35.89	36.62	36.14	P	P	P

Values used for Fig 2C. UD, undetermined; P, positive; N, negative

[a] [1] "SARS-CoV-2 strain BetaCoV/Munich/ChVir984/2020 provided by the Charité-Universitätsmedizin Berlin Institute of Virology, Berlin, Germany; human coronaviruses HCoV-NL63 and HCoV-OC43 cultivated by the University Medical Center Groningen, Groningen, the Netherlands, and sample specifications selected based on past performance data from regular provided coronavirus EQA schemes; negative control sample contained transport medium only (which was used as sample matrix for the EQA samples)."

[b] Values obtained using ddPCR assay (see Materials and Methods for details). [1]: "Samples CVOP20S-07, -01, -03 and - 08 are in a calibrated dilution series. CVOP20S-06 is a duplicate sample of CVOP20S-01. The values provided are for reference only."

1. Matheeussen V, Corman VM, Donoso Mantke O, McCulloch E, Lammens C, Goossens H, et al. International external quality assessment for SARS-CoV-2 molecular detection and survey on clinical laboratory preparedness during the COVID-19 pandemic, April/May 2020. Euro Surveill. 2020;25(27). Epub 2020/07/17. doi: 10.2807/1560-7917.ES.2020.25.27.2001223. PubMed PMID: 32672149; PubMed Central PMCID: PMCPMC7364759.
