# Supplementary material for: A sensitive and affordable multiplex RT-qPCR assay for SARS-CoV-2 detection
Source: PLoS Biol. 2020 Dec 15;18(12):e3001030. doi: 10.1371/journal.pbio.3001030 (PMC7771873; doi:10.1371/journal.pbio.3001030)
Supplement: S4 Table — Values used for Figs 3 and 4 and S2 and S4 Figs. (PDF) [file pbio.3001030.s004.pdf]

S4 Table. The N1E-RP and N2E-RP 4-plex assays perform as well as the TaqPath CE-IVD assay on patient samples

	N1E-RP assay				N2E-RP assay				TaqPath assay				Conclusion		
**Patient**	**N1**	**E**	**RPP30**	**PhHV**	**N2**	**E**	**RPP30**	**PhHV**	**N**	**ORF1ab**	**S**	**MS2**	**N1E-RP**	**N2E-RP**	**TaqPath**
P20	UD	UD	25.56	30.74	UD	UD	25.25	31.41	UD	UD	UD	24.31	N	N	N
P21	UD	UD	29.53	34.27	UD	UD	29.76	34.64	UD	UD	UD	26.50	N	N	N
P22	UD	UD	24.59	33.10	UD	UD	24.47	34.14	UD	UD	UD	25.01	N	N	N
P23	UD	UD	30.71	33.47	UD	UD	30.53	33.93	UD	UD	UD	25.90	N	N	N
P24	UD	UD	30.67	33.89	UD	UD	30.57	34.87	UD	UD	UD	26.04	N	N	N
P25	UD	UD	26.81	32.22	UD	UD	26.86	32.71	UD	UD	UD	24.34	N	N	N
P26	UD	UD	26.52	32.60	UD	UD	26.60	33.39	UD	UD	UD	24.22	N	N	N
P27	UD	UD	24.72	32.38	UD	UD	24.83	33.63	UD	UD	UD	25.62	N	N	N
P28	UD	UD	29.83	34.63	UD	UD	29.98	37.73	UD	UD	UD	25.68	N	N	N
P29	UD	UD	25.23	32.30	UD	UD	25.55	32.94	UD	UD	UD	24.59	N	N	N
P30	39.97	UD	26.45	32.73	UD	UD	26.92	33.71	UD	UD	UD	25.62	N	N	N
P31	UD	UD	29.77	33.12	UD	UD	30.10	33.58	UD	UD	UD	25.23	N	N	N
P32	UD	UD	26.34	32.17	UD	UD	26.36	32.39	UD	UD	UD	24.51	N	N	N
P33	UD	UD	24.60	32.81	UD	UD	24.63	33.31	UD	UD	UD	25.25	N	N	N
P34	UD	UD	31.59	34.52	UD	UD	31.11	34.22	UD	UD	UD	25.43	N	N	N
P35	UD	UD	23.00	33.15	UD	UD	22.77	33.35	UD	UD	UD	25.97	N	N	N
P36	UD	UD	25.61	30.87	UD	UD	26.31	32.66	UD	UD	UD	24.95	N	N	N
P37	UD	UD	29.57	34.96	UD	UD	29.28	35.14	UD	UD	UD	25.78	N	N	N
P38	UD	UD	31.26	34.22	UD	UD	31.14	34.67	UD	UD	UD	26.81	N	N	N
P39	UD	UD	21.62	31.94	UD	UD	21.50	32.33	UD	UD	UD	24.79	N	N	N
P40	UD	UD	30.16	33.34	UD	UD	29.42	33.91	UD	UD	UD	25.82	N	N	N
P41	UD	UD	22.61	32.60	UD	UD	22.49	33.39	UD	UD	UD	25.45	N	N	N
P42	UD	UD	21.32	31.32	UD	UD	21.16	32.45	UD	UD	UD	25.23	N	N	N
P43	UD	UD	27.02	32.57	UD	UD	26.31	32.17	UD	UD	UD	25.01	N	N	N

	N1E-RP assay				N2E-RP assay				TaqPath assay				Conclusion		
**Patient**	**N1**	**E**	**RPP30**	**PhHV**	**N2**	**E**	**RPP30**	**PhHV**	**N**	**ORF1ab**	**S**	**MS2**	**N1E-RP**	**N2E-RP**	**TaqPath**
P44	34.26	31.95	24.16	31.99	33.42	32.56	23.96	32.50	32.77	34.76	33.12	25.17	P	P	P
P45	39.35	UD	30.63	33.82	UD	UD	30.75	35.61	UD	UD	UD	27.19	N	N	N
P46	UD	UD	21.65	31.33	UD	UD	21.68	32.11	UD	UD	UD	24.49	N	N	N
P47	UD	UD	24.73	33.07	UD	UD	24.46	33.90	UD	UD	UD	25.31	N	N	N
P48	UD	UD	24.83	32.74	UD	UD	24.55	33.01	UD	UD	UD	25.82	N	N	N
P49	UD	UD	29.62	33.85	UD	UD	29.32	34.36	UD	UD	UD	26.10	N	N	N
P50	UD	UD	24.81	32.67	UD	UD	24.68	33.23	UD	UD	UD	26.37	N	N	N
P51	UD	UD	30.65	34.44	UD	UD	30.64	37.60	UD	UD	UD	26.99	N	N	N
P52	25.95	24.70	27.20	31.59	25.63	25.35	26.66	32.75	24.55	25.08	25.14	25.97	P	P	P
P53	36.96	37.19	25.79	31.76	36.93	37.77	25.43	32.55	UD	UD	UD	25.63	P	P	N
P54	UD	UD	25.89	32.51	UD	UD	25.75	33.36	UD	UD	UD	25.25	N	N	N
P55	UD	UD	23.41	31.87	UD	UD	23.54	32.70	UD	UD	UD	25.31	N	N	N
P56	UD	UD	28.33	32.86	UD	UD	28.41	33.26	UD	UD	UD	25.66	N	N	N
P57	UD	UD	28.34	32.90	UD	UD	28.40	33.24	UD	UD	UD	26.14	N	N	N
P58	33.38	32.39	30.65	33.38	34.07	33.65	30.10	34.12	33.29	37.49	34.89	26.82	P	P	P
P59	39.03	UD	28.94	32.99	UD	UD	28.64	33.57	UD	UD	UD	25.77	N	N	N
P60	39.53	UD	27.94	31.77	UD	UD	27.67	32.46	UD	UD	UD	25.11	N	N	N
P61	UD	UD	26.81	31.93	UD	UD	26.65	32.29	UD	UD	UD	24.90	N	N	N
P62	UD	UD	32.13	36.32	UD	UD	31.71	36.92	UD	UD	UD	27.27	N	N	N
P63	UD	UD	28.42	32.91	UD	UD	27.90	33.90	UD	UD	UD	25.95	N	N	N
P64	UD	UD	31.67	34.94	UD	UD	31.54	35.91	UD	UD	UD	27.53	N	N	N
P65	UD	UD	22.81	32.49	UD	UD	22.49	33.30	UD	UD	UD	25.98	N	N	N
P66	29.86	29.45	22.54	31.03	30.72	30.51	22.53	31.77	29.45	30.68	30.76	24.86	P	P	P
P67	32.66	31.37	24.95	31.89	32.65	32.39	25.25	32.77	32.25	34.90	33.81	25.78	P	P	P
P68	UD	UD	24.51	32.57	UD	UD	24.28	33.37	UD	UD	UD	25.67	N	N	N
P69	UD	UD	26.49	33.05	UD	UD	26.57	34.02	UD	UD	UD	25.48	N	N	N

	N1E-RP assay				N2E-RP assay				TaqPath assay				Conclusion		
**Patient**	**N1**	**E**	**RPP30**	**PhHV**	**N2**	**E**	**RPP30**	**PhHV**	**N**	**ORF1ab**	**S**	**MS2**	**N1E-RP**	**N2E-RP**	**TaqPath**
P70	UD	UD	24.67	31.73	UD	UD	24.43	32.22	UD	UD	UD	24.40	N	N	N
P71	40.23	UD	20.35	30.96	UD	UD	20.32	31.66	UD	UD	UD	24.65	N	N	N
P72	43.15	UD	22.83	32.07	UD	UD	22.59	32.43	UD	UD	UD	25.15	N	N	N
P73	UD	UD	28.21	33.01	UD	UD	28.13	33.57	UD	UD	UD	25.95	N	N	N
P74	UD	UD	25.78	32.16	UD	UD	25.66	32.53	UD	UD	UD	24.71	N	N	N
P75	38.44	35.68	26.65	32.44	39.20	38.93	26.31	33.25	35.47	UD	UD	25.79	P	P	Inc
P76	22.69	22.62	25.77	32.01	23.04	23.42	25.12	32.39	21.91	22.55	22.77	26.69	P	P	P
P77	21.47	21.60	23.90	33.21	21.84	22.33	23.98	32.68	21.16	22.53	22.38	30.08	P	P	P
P78	UD	UD	24.49	32.73	UD	UD	24.37	33.07	UD	UD	UD	25.72	N	N	N
P79	UD	UD	27.14	33.85	UD	UD	27.06	35.00	UD	UD	UD	26.04	N	N	N
P80	UD	UD	22.78	32.70	UD	UD	22.89	33.24	UD	UD	UD	25.80	N	N	N
P81	UD	UD	23.01	32.61	UD	UD	22.74	32.79	UD	UD	UD	25.57	N	N	N
P82	UD	UD	26.99	31.79	UD	UD	26.84	32.72	UD	UD	UD	25.52	N	N	N
P83	UD	UD	31.29	35.55	UD	UD	31.42	35.57	UD	UD	UD	27.24	N	N	N
P84	UD	UD	22.18	31.85	UD	UD	21.99	32.82	UD	UD	UD	24.75	N	N	N
P85	UD	UD	24.37	32.50	UD	UD	24.14	33.25	UD	UD	UD	25.85	N	N	N
P86	UD	UD	23.23	31.79	UD	UD	23.15	32.62	UD	UD	UD	24.80	N	N	N
P87	UD	UD	23.10	31.79	44.55	UD	22.99	31.89	UD	UD	UD	24.76	N	N	N
P88	UD	UD	24.51	32.62	UD	UD	24.48	33.49	UD	UD	UD	25.17	N	N	N
P89	UD	UD	24.67	32.02	UD	UD	24.45	34.68	UD	UD	UD	25.17	N	N	N
P90	25.58	25.96	23.32	31.65	26.04	26.56	23.27	32.18	24.15	24.71	24.87	24.73	P	P	P
P91	UD	UD	20.27	31.55	UD	UD	20.30	31.207	UD	UD	UD	24.95	N	N	N
P92	17.51	16.25	20.36	29.33	17.24	16.79	20.49	31.68	17.28	16.97	17.10	27.18	P	P	P
P93	UD	UD	22.97	32.74	UD	UD	22.89	33.44	UD	UD	UD	25.60	N	N	N
P94	UD	UD	23.60	32.46	UD	UD	23.70	33.29	UD	UD	UD	25.16	N	N	N
P95	UD	UD	25.18	32.93	UD	UD	24.99	33.28	UD	UD	UD	26.64	N	N	N

	N1E-RP assay				N2E-RP assay				TaqPath assay				Conclusion		
**Patient**	**N1**	**E**	**RPP30**	**PhHV**	**N2**	**E**	**RPP30**	**PhHV**	**N**	**ORF1ab**	**S**	**MS2**	**N1E-RP**	**N2E-RP**	**TaqPath**
P96	UD	UD	23.53	33.50	UD	UD	23.31	34.08	UD	UD	UD	25.88	N	N	N
P97	UD	UD	25.64	31.92	UD	UD	25.09	31.82	UD	UD	UD	24.83	N	N	N
P98	21.50	20.39	23.62	31.84	21.29	20.90	23.20	32.92	20.73	20.43	21.33	25.97	P	P	P
P99	UD	UD	23.01	32.16	UD	UD	23.43	34.11	UD	UD	UD	27.60	N	N	N
P100	18.24	18.92	21.51	32.04	18.68	19.44	21.99	32.50	17.91	18.05	18.69	25.49	P	P	P
P101	UD	UD	23.77	32.02	UD	UD	24.05	32.62	UD	UD	UD	25.25	N	N	N
P102	UD	UD	24.71	30.80	UD	UD	24.59	31.29	UD	UD	UD	24.31	N	N	N
P103	UD	UD	23.61	32.64	UD	UD	23.55	32.82	UD	UD	UD	25.13	N	N	N
P104	UD	UD	23.12	31.92	UD	UD	22.77	32.44	UD	UD	UD	24.57	N	N	N
P105	24.75	25.23	25.61	31.03	25.26	25.85	25.33	31.82	25.95	26.11	26.42	24.74	P	P	P
P106	UD	UD	26.67	32.93	UD	UD	26.60	32.68	UD	UD	UD	25.36	N	N	N
P107	33.90	32.43	21.76	31.97	34.09	33.04	21.71	31.89	32.29	33.97	32.74	24.80	P	P	P
P108	UD	UD	27.70	32.01	UD	UD	27.35	33.17	UD	UD	UD	24.69	N	N	N
**Controls**															
-ve (extr)	UD	UD	UD	31.75	UD	UD	UD	32.77	UD	UD	UD	25.18	OK	OK	OK
-ve	UD	UD	UD	UD	UD	UD	UD	UD	UD	UD	UD	UD	OK	OK	OK
+ve	30.89	34.15	*	UD	30.35	34.49	*	UD	31.28	31.88	31.23	UD	OK	OK	OK

Values used for Figs 3 and 4; S2 and S4 Figs. UD, undetermined; P, positive; N, negative; Inc, inconclusive

-ve (extr), negative control with viral transport medium after RNA isolation (does not contain SARS-CoV-2 or human material; does contain PhHV);

-ve, negative control containing water only (should not contain any RNA)

+ve, positive control with in vitro transcribed RNA (50 copies; contains SARS-CoV-2 target RNA, does not contain human or PhHV RNA)

* UD, but FAM-positive samples give ~5% signal bleed through into the JOE channel; this should be taken into account for samples without any true RPP30 (HEX) signal
