# Supplementary material for: A sensitive and affordable multiplex RT-qPCR assay for SARS-CoV-2 detection
Source: PLoS Biol. 2020 Dec 15;18(12):e3001030. doi: 10.1371/journal.pbio.3001030 (PMC7771873; doi:10.1371/journal.pbio.3001030)
Supplement: S5 Table — High-quality genome sequences analysed only (n = 97,782). RdRp, RNA-dependent RNA polymerase; SARS-CoV-2, Severe Acute Respiratory Syndrome Coronavirus 2. (PDF) [file pbio.3001030.s005.pdf]

S5 Table. Percentage of known SARS-CoV-2 genomic sequences with mutations in primer/probe binding sites for RdRp, M and S gene assays

Assay	Primer/probe	Percentage of strains with any mismatch/deletion [a]		Percentage of strains with mismatch in 5 most 3' nt [a]	
		Per primer/probe	Per assay [b]	Per primer	Per assay [c]
RdRp	RdRp_SARSr-F2	0.44%	100%	0.071%	0.078%
	RdRp_SARSr-P2	0.66%		(0.005%)	
	RdRp_SARSr-R1	100%		0.007%	
M gene	M-475-F	0.16%	1.11%	0.042%	0.045%
	M-507-P	0.87%		(0.857%)	
	M-574-R	0.08%		0.003%	
S gene	S-Gene-F	0.65%	1.91%	0.067%	0.375%
	S-Gene-P	0.74% (0.31%) [a]		(0.043%)	
	S-Gene-R	0.55%		0.308%	

High quality genome sequences analysed only (n=97,782); also see S1 Data.

[a] No genomes have deletions in primer/probe binding regions, with the exception of the S-gene probe, which has a 6 nt deletion in 0.31% of genome sequences. Presence of these changes does not necessarily impact on primer/probe performance. Mutations at the 3’ ends for the primer regions (here defined as the final 5 nt) are more likely to affect assay sensitivity.

[b] Mismatches/deletions in the primer/probe set used in single target assay

[c] Mismatches in the forward and reverse primers only. For probes values are given between brackets for reference (in the “per primer” column).
