# Supplementary material for: A sensitive and affordable multiplex RT-qPCR assay for SARS-CoV-2 detection
Source: PLoS Biol. 2020 Dec 15;18(12):e3001030. doi: 10.1371/journal.pbio.3001030 (PMC7771873; doi:10.1371/journal.pbio.3001030)
Supplement: S6 Table — qRT-PCR, quantitative reverse transcription PCR. (PDF) [file pbio.3001030.s006.pdf]

S6 Table. Interpretation and suggested action based on N1E-RP or N2E-RP qRT-PCR results

SARS-CoV-2 targets	RPP30 Cq	Interpretation	Suggested action
No positives (UD, or Cq > 40)	High*	Inconclusive (possible negative), but poor sample	Repeat sampling
	Low*	Negative	Report negative
One weak positive (Cq 35-40)	High	Inconclusive; poor sample	Repeat sampling
	Low	Inconclusive	Perform different assay with different primers/probes and of equal or higher sensitivity
Two weak positives (Cq 35-40)	High	Positive; poor sample	Report positive
	Low	Likely (weak) positive	Report positive
One moderate positive (Cq 30-35)	High	Inconclusive, likely positive; poor sample	Perform different assay with different primers/probes and of equal or higher sensitivity (consider repeat sampling)
	Low	Inconclusive, possible positive	Perform different assay with different primers/probes and of equal or higher sensitivity
Two moderate positives (Cq 30-35)	High	Positive; poor sample, but does not affect conclusion	Report positive
	Low	Positive	Report positive
One strong positive (Cq < 30)	High	Inconclusive, likely positive; poor sample	Perform different assay with different primers/probes and of equal or higher sensitivity (consider repeat sampling)
	Low	Inconclusive, likely positive (second target may have mutations)	Perform different assay with different primers/probes and of equal or higher sensitivity to rule out false positive
Two strong positives (Cq < 30)	High	Positive; poor sample, but does not affect conclusion	Report positive
	Low	Positive	Report positive

High PhHV Cq values (>35; but depending on the exact amount used for spike-in; 2 to 3 cycles above the expected Cq is a good guide) indicate poor RNA isolation and/or presence of PCR inhibitors. Therefore, any negative (i.e. both SARS-CoV-2 targets not detected) or inconclusive samples (i.e. only one SARS-CoV-2 targets detected) should be interpreted in this context, and repeat sampling/testing must be performed. This has no impact on positive samples (both SARS-CoV-2 targets detected), which should still be reported as such.

* The precise RPP30 Cq cut-off value remains to be determined. This requires collection of a much larger amount of data, including repeat sampling of sufficient numbers of patients with varying RPP30 Cq values and initial negative test results. As an initial guide repeat sampling could be performed for any sample with RPP30 Cq >30. For any samples with RPP30 Cq values undetermined or >40, repeat sampling is essential, unless both SARS-CoV-2 targets are <40, in which case the test result is positive.
