# Supplementary material for: A sensitive and affordable multiplex RT-qPCR assay for SARS-CoV-2 detection
Source: PLoS Biol. 2020 Dec 15;18(12):e3001030. doi: 10.1371/journal.pbio.3001030 (PMC7771873; doi:10.1371/journal.pbio.3001030)
Supplement: S1 Fig — N1E-RP and N2E-RP RT-qPCR assays were performed on (A) 1 to 10,000 copies of SARS-CoV-2 control RNA (IVT) before (as Fig 2A) and after nucleic acid extraction, (B) a serial dilution of RNA isolated from cultured SARS-CoV-2, before (as Fig 2B) and after re-extraction. Mean ± SD for technical triplicates; R2 values for logarithmic trend line fitting and amplification efficiencies (E) for samples after (re)extraction. Also, see S1 and S2 Tables and S1 Data. IVT, in vitro transcribed; SARS-CoV-2, Severe Acute Respiratory Syndrome Coronavirus 2; SD, standard deviation. (PDF) [file pbio.3001030.s007.pdf]

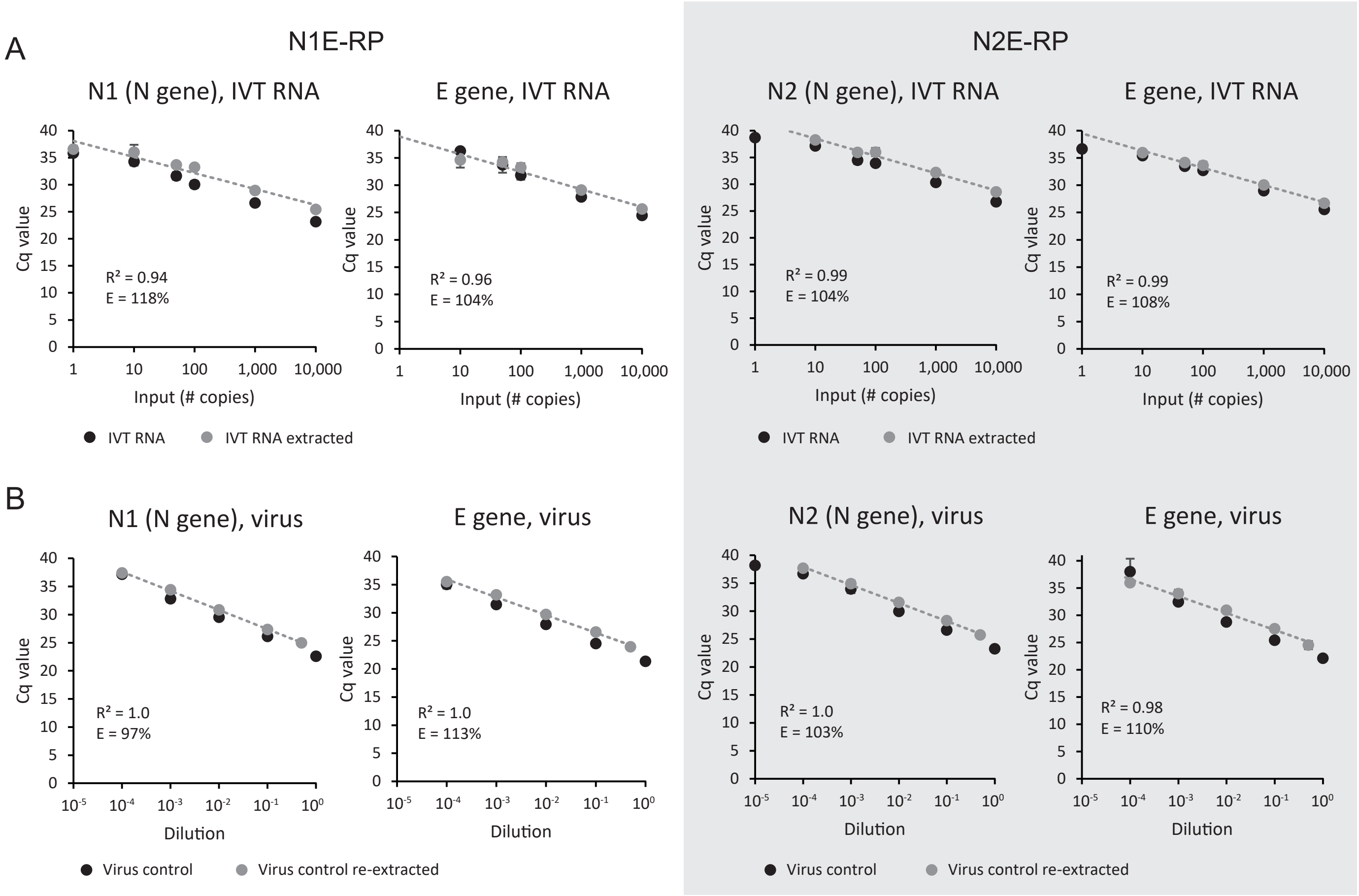

S1 Fig. RNA extraction has no substantial impact on the sensitivity of the N1E-RP and N2E-RP 4-plex assays. N1E-RP and N2E-RP RT-qPCR assays were performed on (A) 1 to 10,000 copies of SARS-CoV-2 control RNA (IVT, in vitro transcribed) before (as Fig 2A) and after nucleic acid extraction, (B) a serial dilution of RNA isolated from cultured SARS-CoV-2, before (as Fig 2B) and after re-extraction. Mean ± SD for technical triplicates; R^2 values for logarithmic trend line fitting and amplification efficiencies (E) for samples after (re)extraction. Also, see Table S1 and S2, and S1 Data.
