# Supplementary material for: A sensitive and affordable multiplex RT-qPCR assay for SARS-CoV-2 detection
Source: PLoS Biol. 2020 Dec 15;18(12):e3001030. doi: 10.1371/journal.pbio.3001030 (PMC7771873; doi:10.1371/journal.pbio.3001030)
Supplement: S2 Fig — (A, B) Cq values for internal controls, MS2 for TaqPath and PhHV for N1E-RP and N2E-RP assays (A), and RPP30 controls (B). (C) Cq values for PhHV and RPP30 controls for N1E-RP and N2E-RP assays, ranked by RPP30 values from the N1E-RP assay, confirm that variability does not substantially correlate with extraction efficiency. Also, see Table 1, S4 Table, and S1 Data. Cq, cycle quantification; NTS, nose and throat swabs; PhHV, Phocine Herpes Virus. (PDF) [file pbio.3001030.s008.pdf]

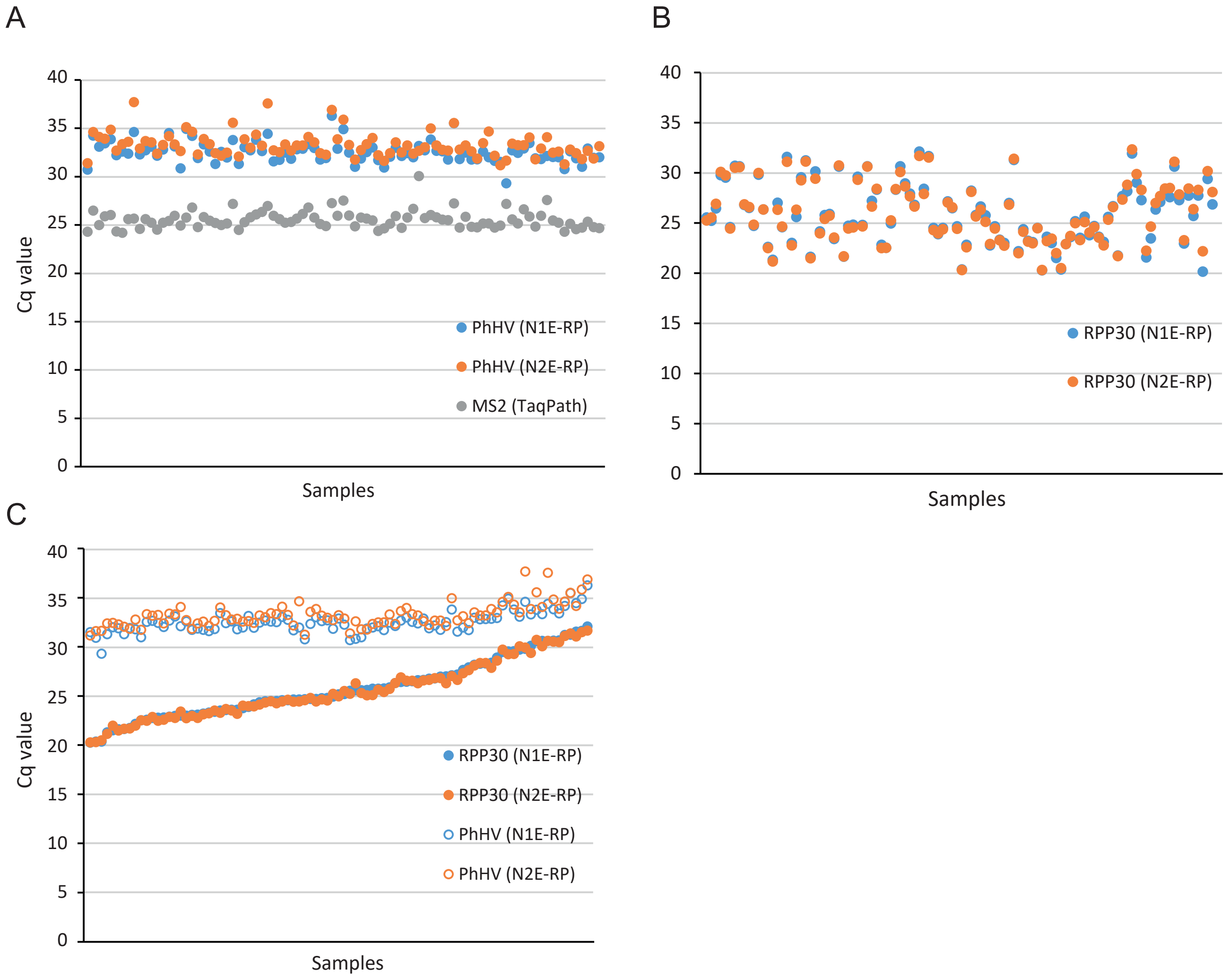

S2 Fig. High reproducibility for extraction controls, but high variability for the human *RPP30* control in NTS samples. (A, B) Cq values for internal controls, MS2 for TaqPath and PhHV for N1E-RP and N2E-RP assays (A), and *RPP30* controls (B). (C) Cq values for PhHV and *RPP30* controls for N1E-RP and N2E-RP assays, ranked by *RPP30* values from the N1E-RP assay, confirm that variability does not substantially correlate with extraction efficiency. Also, see Table 1, S4 Table, and S1 Data.
