# Supplementary material for: A sensitive and affordable multiplex RT-qPCR assay for SARS-CoV-2 detection
Source: PLoS Biol. 2020 Dec 15;18(12):e3001030. doi: 10.1371/journal.pbio.3001030 (PMC7771873; doi:10.1371/journal.pbio.3001030)
Supplement: S3 Fig — (A) Cq values for RPP30 on a serial dilution of positive control plasmid DNA (100,000 down to 10 copies were tested). (B) Cq values for RPP30 on NAs isolated from human cultured cells (1 = undiluted) and NA isolated from a serial dilution of the same cell suspension show a strong linear correlation and 92% amplification efficiency. Negative control samples did not show any amplification. Data points and error bars, mean ± SD (n = 2 technical replicates). R2 values for logarithmic trend line fitting; E, amplification efficiency. Also, see S1 Data. Cq, cycle quantification; NA, nucleic acid; SD, standard deviation. (PDF) [file pbio.3001030.s009.pdf]

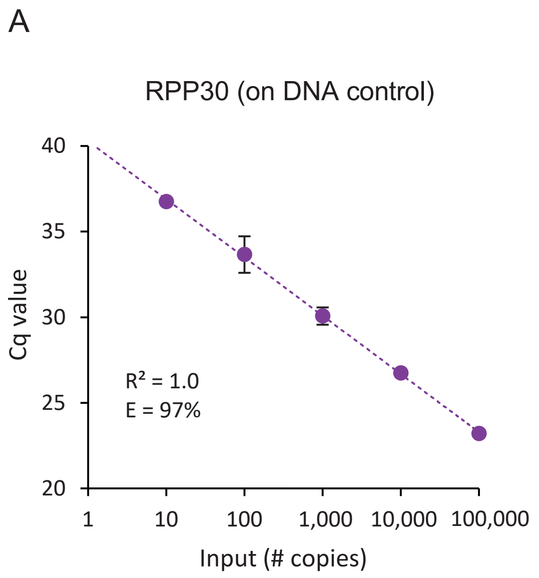

S3 Fig. The human *RPP30* control probe can detect 10 copies of control DNA. (A) Cq values for *RPP30* on a serial dilution of positive control plasmid DNA (100,000 down to 10 copies were tested). (B) Cq values for *RPP30* on nucleic acids (NA) isolated from human cultured cells (1 = undiluted) and NA isolated from a serial dilution of the same cell suspension show a strong linear correlation and 92% amplification efficiency (E). Negative control samples did not show any amplification. Data points and error bars, mean ± SD (n = 2 technical replicates). R^2 values for logarithmic trend line fitting; E, amplification efficiency. Also, see S1 Data.
