# Supplementary material for: A sensitive and affordable multiplex RT-qPCR assay for SARS-CoV-2 detection
Source: PLoS Biol. 2020 Dec 15;18(12):e3001030. doi: 10.1371/journal.pbio.3001030 (PMC7771873; doi:10.1371/journal.pbio.3001030)
Supplement: S4 Fig — (A, B) Comparison of actual (Act) and RPP30-normalised (Norm) Cq values for SARS-CoV-2 targets shows that values cluster more strongly after normalisation, both when normalising to the worst (highest RPP30 Cq; A) and best (lowest RPP30 Cq; B). Increased clustering (i.e., reduced variability) demonstrates the impact of the correlation between quantity of human and viral nucleic acids in NTS samples (Fig 4C). This shows (1) that sample quality substantially impacts on assay sensitivity; and (2) that variability in viral loads is smaller than non-normalised data suggest. Plots in (A) demonstrate that 4–6 positives (15%–23%) would likely have been below the detection limit (above the red line, viral Cq > 40) in a worst quality sample scenario. Also, see Table 1, S4 Table, and S1 Data. Cq, cycle quantification; NTS, nose and throat swabs; SARS-CoV-2, Severe Acute Respiratory Syndrome Coronavirus 2 (PDF) [file pbio.3001030.s010.pdf]

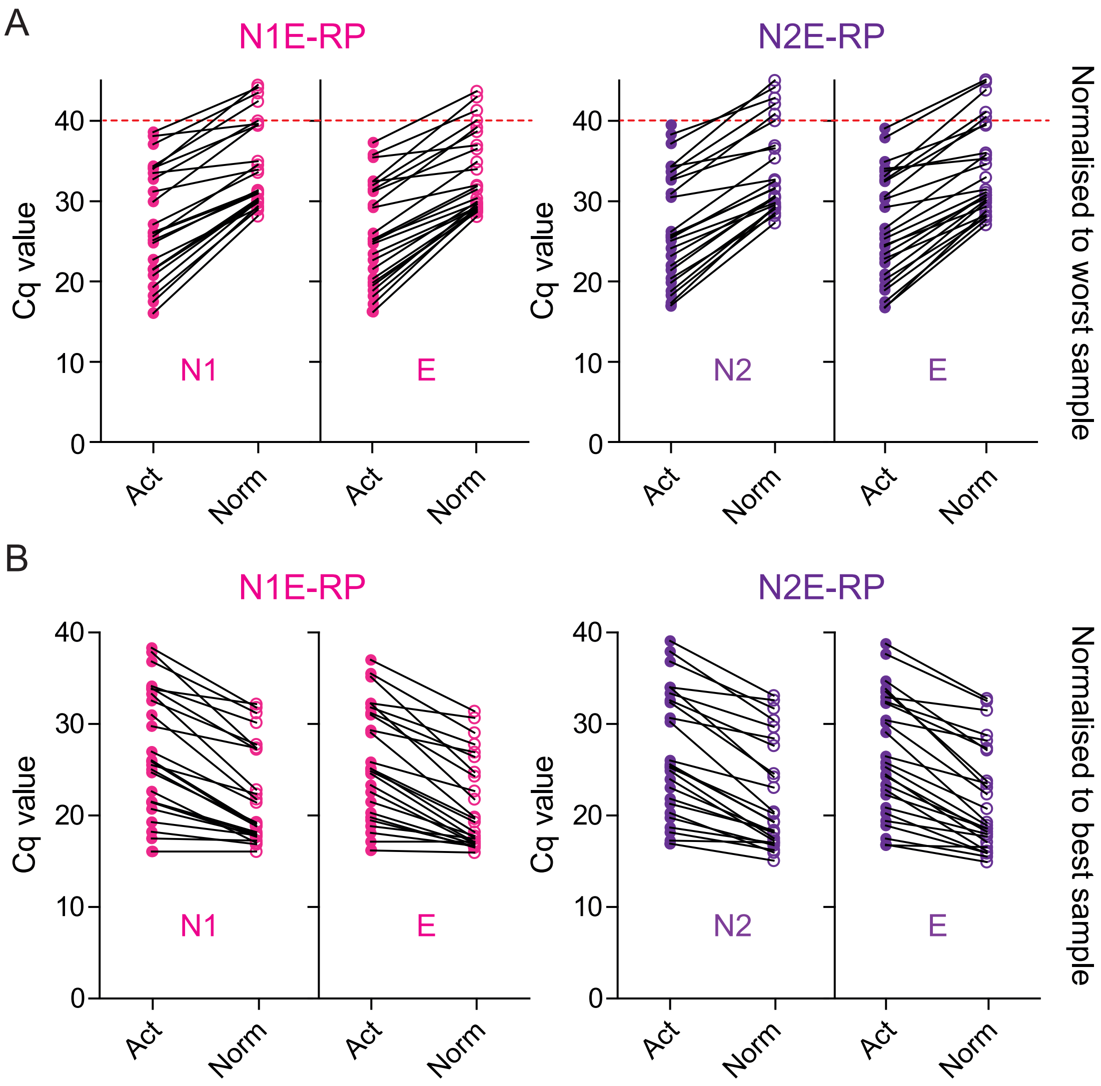

S4 Fig. Increased chance of false negatives for low quality NTS samples, with high *RPP30* Cq values. (A, B) Comparison of actual (Act) and *RPP30*-normalised (Norm) Cq values for SARS-CoV-2 targets shows that values cluster more strongly after normalisation, both when normalising to the worst (highest *RPP30* Cq; A) and best (lowest *RPP30* Cq; B). Increased clustering (i.e. reduced variability) demonstrates the impact of the correlation between quantity of human and viral nucleic acids in NTS samples (Fig 4C). This shows 1) that sample quality substantially impacts on assay sensitivity and 2) that variability in viral loads is smaller than non-normalised data suggest. Plots in (A) demonstrate that 4-6 positives (15-23%) would likely have been below the detection limit (above the red line, viral Cq > 40) in a worst quality sample scenario. Also, see Table 1, S4 Table, and S1 Data.
