# Supplementary material for: A sensitive and affordable multiplex RT-qPCR assay for SARS-CoV-2 detection
Source: PLoS Biol. 2020 Dec 15;18(12):e3001030. doi: 10.1371/journal.pbio.3001030 (PMC7771873; doi:10.1371/journal.pbio.3001030)
Supplement: S1 Protocol — RT-qPCR, quantitative reverse transcription PCR; SARS-CoV-2, Severe Acute Respiratory Syndrome Coronavirus 2. (PDF) [file pbio.3001030.s011.pdf]

S1 Protocol: 4-plex SARS-CoV-2 RT-qPCR assays

Primers/probes

Dissolve primers and probes (Table P1; HPLC purified) in 10 mM Tris, 0.1 mM EDTA, pH 8.0 (IDTE Cat. No. 11-05-01-09, or similar) for 100 µM stocks. Prepare primer/probe mixes (Tables P2-P7), and store aliquots at -20°C. Working stocks can be stored at 4°C.

Table P1. Primer/Probe details for 4-plex assays

Target	Oligonucleotide ID	Sequence (5'-3')	PCR Product Size	Reference
SARS-CoV-2, E gene	E_Sarbeco_F1	ACAGGTACGTTAATAGTTAATAGCGT	113 bp	[1, 2]
	E_Sarbeco_R2	ATATTGCAGCAGTACGCACACA		
	TxRd_E_Sarbeco_P1*	CFR-610-ACACTAGCCATCCTTACTGCGCTTCG-BHQ2		
SARS-CoV-2, N gene	2019-nCoV_N1-F	GACCCCAAAATCAGCGAAAT	72 bp	[3]
	2019-nCoV_N1-R	TCTGGTTACTGCCAGTTGAATCTG		
	2019-nCoV_N1-P	FAM-ACCCCGCATTACGTTTGGTGGACC-BHQ1		
SARS-CoV-2, N gene	2019-nCoV_N2-F	TTACAAACATTGGCCGCAAA	67 bp	[3]
	2019-nCoV_N2-R	GCGCGACATTCCGAAGAA		
	2019-nCoV_N2-P	FAM-ACAATTTGCCCCCAGCGCTTCAG-BHQ1		
Human *RPP30*	Hs_RPP30-F	AGATTTGGACCTGCGAGCG	65 bp	[3]
	Hs_RPP30-R	GAGCGGCTGTCTCCACAAGT		
	HEX-Hs_RPP30-P	HEX–TTCTGACCTGAAGGCTCTGCGCG–BHQ1		
PhHV-1, Glycoprotein B	PhHV-F	GGGCGAATCACAGATTGAATC	89 bp	[4]
	PhHV-R**	GCGGTTCCAAACGTACCA(A)		
	Cy5-PhHV-P***	Quasar-670-TTTTTATGTGTCCGCCACCATCTGGATC-BHQ2		

* Probe named TxRd for simplicity, CAL Flour Red (CFR-610) has virtually identical properties to TexRed

** Reverse primer GCGGTTCCAAACGTACCA used for our work; GCGGTTCCAAACGTACCAA used in [4]; both should work equally

*** Probe named Cy5 for simplicity. Quasar 670 has virtually identical properties to Cy5

Primer/Probe Mixes

Table P2. 50x N1 (FAM) Primer/Probe Mix

Oligonucleotide	Stock Concentration (µM)	Concentration in 50x mix (µM)	Concentration in reaction (nM)	Volume (µL)
2019-nCoV_N1-F	100	25	500	50
2019-nCoV_N1-R	100	25	500	50
2019-nCoV_N1-P	100	6.25	125	12.5
Nuclease free water	-	-	-	87.5
**Total**				**200**

Table P3. 50x N2 (FAM) Primer/Probe Mix

Oligonucleotide	Stock Concentration (µM)	Concentration in 50x mix (µM)	Concentration in reaction (nM)	Volume (µL)
2019-nCoV_N2-F	100	25	500	50
2019-nCoV_N2-R	100	25	500	50
2019-nCoV_N2-P	100	6.25	125	12.5
Nuclease free water	-	-	-	87.5
**Total**				**200**

Table P4. 50x E gene (TexRed) Primer/Probe Mix

Oligonucleotide	Stock Concentration (µM)	Concentration in 50x mix (µM)	Concentration in reaction (nM)	Volume (µL)
E_Sarbeco_F1	100	20	400	40
E_Sarbeco_R2	100	20	400	40
TxRd_E_Sarbeco_P1	100	10	200	20
Nuclease free water	-	-	-	100
**Total**				**200**

Table P5. 50x *RPP30* (HEX) Primer/Probe Mix

Oligonucleotide	Stock Concentration (µM)	Concentration in 50x mix (µM)	Concentration in reaction (nM)	Volume (µL)
Hs_RPP30-F	100	25	500	50
Hs_RPP30-R	100	25	500	50
HEX-Hs_RPP30-P	100	6.25	125	12.5
Nuclease free water	-	-	-	87.5
**Total**				**200**

Table P6. 50x PhHV (Cy5) Primer/Probe Mix

Oligonucleotide	Stock Concentration (µM)	Concentration in 50x mix (µM)	Concentration in reaction (nM)	Volume (µL)
PhHV-F	100	15	300	30
PhHV-R	100	15	300	30
Cy5-PhHV-P	100	5	100	10
Nuclease free water	-	-	-	130
**Total**				**200**

Table P7. 12.5x 4-plex Primer/Probe Mix (for 100 reactions)

	Stock	Probe Concentration in 12.5x mix (µM)	Probe Concentration in reaction (nM)	Volume (µL)
N1 or N2 mix (FAM)	50x	1.56	125	50
E mix (TxRed)	50x	2.5	200	50
RPP30 mix (HEX)	50x	1.56	125	50
PhHV mix (Cy5)	50x	1.25	100	50
**Total**				**200**

RT-qPCR

Any Real-Time qPCR machine that can detect the four different channels and has been calibrated for the appropriate fluorophores can be used. We use the Applied Biosystems™ 7500 Fast Real-Time PCR Systems (channels FAM, JOE, TEXAS RED and CY5) and 7500 Software v2.3, MicroAmp Fast Optical 0.1mL 96-well reaction plates (Cat. No. 4346906) and Optical Adhesive film (Cat. No. 4311971). We performed all RT-qPCRs with Takara One Step PrimeScript™ III RT-qPCR kit (Cat. No. RR600B). Other One-Step mixes are available, but may require slightly different reaction conditions and may change sensitivity of SARS-CoV-2 detection.

N1E-RP and N2E-RP 4-plex assay

Master Mix (20 µl per reaction), assemble on ice

H_2O (RNase free)	5.5 µl	for 100x	550 µl
2x reaction mix	12.5 µl		1250 µl
12.5x primer/probe mix	2 µl		200 µl

Mix with template RNA 5 µl

Spin down before transferring to PCR machine

PCR program

52°C	5 min (reverse transcription)		
95°C	10 s (RT inactivation/denaturation)		
45 cycles of		95°C	3 s (denaturation)
		60°C	30 s (amplification & detection)

Controls

Add the viral spike-in control to the lysis buffer master mix before sample inactivation.

[For our assays, we used 25 µl of culture supernatant containing PhHV particles to 25 ml of lysis buffer. This amount was previously shown to give a Cq value of ~33.]

For each plate, include the following controls:

H10, negative extraction control, VTM extracted;

H11, non-extraction negative control, water only;

H12, 50 copies of positive control RNA (see below).

Positive control RNA

Positive control RNAs generated by in vitro transcription (IVT) were provided by:

- Sylvie Behillil (Institut Pasteur, Paris, France): mix of E gene and RdRp (the latter is <u>not</u> the same as the Corman et al (2020) RdRp template) [1-3] at 10^9 copies/µl.

- Christine Tait-Burkard (Roslin Institute, Edinburgh, UK): RdRp [1, 2], N1/N3 and N2 [3]. Three individual IVT RNAs at known concentrations (ng/µl) were provided; molecular weights used to determine the concentrations in copies/µl and 10^9 copies/µl prepared for each RNA.

Prepare all stocks and dilutions in Eppendorf DNA LoBind tubes (Cat. No. 10051232).

1. Prepare an equimolar mix of all RNAs at 2.5 x 10^8 copies/µl. Store aliquots of this solution at -80°C.

2. Prepare 10^4 copies/µl positive RNA controls and store 5 µl aliquots at -80°C for single use per plate. Mix 5µl of 2.5 x 10^8 copies/µl with 620 µl of water to give 2 x 10^6 copies/µl. Dilute this 20 µl plus 180 µl water giving a 2 x 10^5 copies/µl solution, and 20 µl plus 380 µl giving 10^4 copies/µl.

3. For each plate, a 25 copies/µl solution is made by diluting the 10^4 copies/µl solution by mixing:

2 µl with 98 µl water, then

12.5 µl of this with 87.5 µl water

Of this 25 copies/µl solution, 2 µl is added to well H12 along with 3 µl of water, to give the 50 copy positive control on each plate.

S1 Protocol References

1. Corman VM, et al. Diagnostic detection of 2019-nCoV by real-time RT-PCR, protocol v2 2020. Available from: https://www.who.int/docs/default-source/coronaviruse/protocol-v2-1.pdf.
2. Corman VM, Landt O, Kaiser M, Molenkamp R, Meijer A, Chu DK, et al. Detection of 2019 novel coronavirus (2019-nCoV) by real-time RT-PCR. Euro Surveill. 2020;25(3). Epub 2020/01/30. doi: 10.2807/1560-7917.ES.2020.25.3.2000045. PubMed PMID: 31992387; PubMed Central PMCID: PMCPMC6988269.
3. WHO in-house assays COVID-19 RT-qPCR 2020. Available from: https://www.who.int/docs/default-source/coronaviruse/whoinhouseassays.pdf?sfvrsn=de3a76aa_2.
4. Stranska R, Schuurman R, de Vos M, van Loon AM. Routine use of a highly automated and internally controlled real-time PCR assay for the diagnosis of herpes simplex and varicella-zoster virus infections. J Clin Virol. 2004;30(1):39-44. Epub 2004/04/10. doi: 10.1016/j.jcv.2003.08.006. PubMed PMID: 15072752.
