# Supplementary material for: A sensitive and affordable multiplex RT-qPCR assay for SARS-CoV-2 detection
Source: PLoS Biol. 2020 Dec 15;18(12):e3001030. doi: 10.1371/journal.pbio.3001030 (PMC7771873; doi:10.1371/journal.pbio.3001030)
Supplement: S2 Protocol — (PDF) [file pbio.3001030.s012.pdf]

S2 Protocol: Viral nucleic acid isolation

In principle, the buffers and solution below can replace those of the equivalent buffers in the Omega Mag-Bind Viral DNA/RNA 96 Kit (Cat. No. M6246). Lysis and wash buffers can either be replaced with guanidine thiocyanate (GnSCN) or guanidine hydrochloride (GnHCl) containing solutions, depending on reagent availability. All performed equally well in preliminary tests. All lysis buffers (Omega TNA, GnSCN Lysis Buffer, GnHCl Lysis Buffer, each with/without isopropanol) were shown to inactivate coronavirus after 15 min incubation (Fig P1). Briefly, to determine whether lysis buffers inactivate coronaviruses, 200 µl CoV 229E-GFP [1, 2] stock ($9.6x10^5$ pfu/ml in DMEM, 10% FCS, 1% NEAA) was mixed with lysis buffer at the recommended ratio (240 µl lysis buffer without isopropanol or 520 µl lysis buffer with isopropanol, i.e. 240 µl buffer and 280 µl isopropanol). For positive infection controls, virus was mixed with 240 or 520 µl medium. All mixes were inverted 8 times and incubated at room temperature for 15 min. Cytotoxic components were then removed by centrifugation at 4°C using Microcon filter columns (Millipore; 30 kDa cut-off), and two 0.5 ml PBS washes, similar to previously described methods [3, 4]. Remaining virus particles were then resuspended in 200 µl DMEM and 50 µl of a 1/100 dilution added to HUH7 cells (a cell line permissive to infection by CoV 229E). Cells were seeded the previous day at $1.8x10^4$/well in a black 96-well plate (Corning), and were at ~80% confluence for infection. Cultures were monitored daily for cell viability, cytopathic effects and GFP expression using microscopy. No significant cell death was observed for any of the samples. Relative fluorescence was measured using a Clariostar BMG Plate Reader at 72 h, with fluorescence for a non-infected control set to zero. No fluorescence was observed for any of the lysis buffer treated samples (Fig P1), and fluorescence microscopy confirmed the absence of GFP positive cells, consistent with complete viral inactivation.

In our preliminary tests, we used the March 2020 version of the protocol provided with the Omega Mag-Bind Viral DNA/RNA 96 Kit to test viral nucleic acid isolation with our own solutions and reagents (see below). We used the Mag-Bind Particles CNR from the Omega kit, and although we have not yet tested this, we expect that these can be replaced by SeraSil-Mag silica beads (Cytiva, cat No. 29357375). The March 2020 protocol is different from the April 2020 Supplementary Protocol provided by Omega. The latter was used in combination with original Omega kit components for all other work presented in our manuscript. We do not see any reasons why our solutions would not work equally well with the April 2020 version of the protocol, but have not tested this. All purifications were carried out using a KingFisher Flex robot (Cat. No. 5400610),

KingFisher Deep well plates (Cat. No. 95040450), KingFisher Flex 96 Deep-Well Tip Combs (Cat. No. A43074) and KingFisher 96 microplates (Cat. No. 97002540). Alternative robots could be used; and manual purifications are also possible.

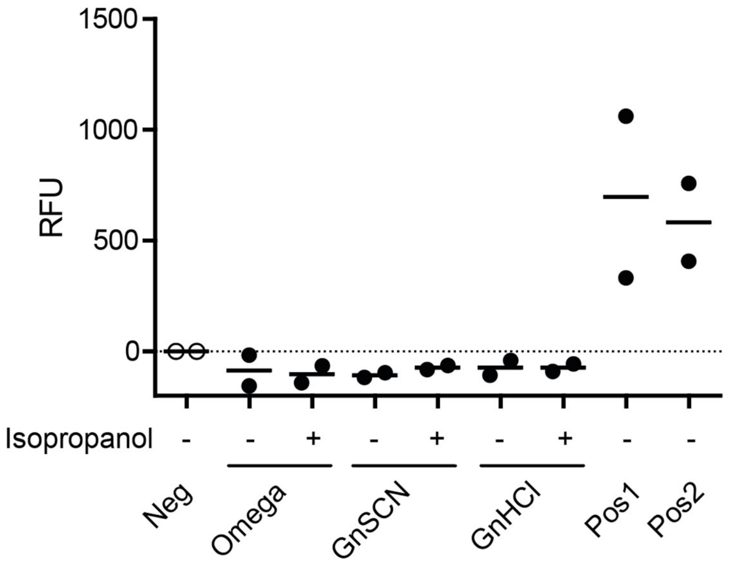

Fig P1. Inactivation of coronavirus CoV-229E by Omega TNA, and GnSCN and GnHCl lysis buffers. CoV 229E-GFP was mixed with lysis buffer (- or + isopropanol) as described and then used to infect HUH7 cells. After 72 h of infection, GFP fluorescence (indicating infected cells) was measured. Negative (Neg) control, no infection; positive control (Pos1 and 2), virus mixed with medium instead of lysis buffer. Fluorescence units (RFU) are expressed relative to background (negative control fluorescence set to 0). Solid lines indicate the mean for n = 2 independent experiments. Also, see S1 Data.

S2 Protocol: Viral nucleic acid isolation

Protocol in brief (tested with home-made solutions; based on Omega March 2020 protocol)

1. Per sample, prepare 528 µl master mix: 240 µl lysis buffer, 8 µl carrier RNA (1 µg/µl) and 280 µl isopropanol
2. Add 200 µl of patient sample in VTM, mix thoroughly; incubate for >15 min
3. Add 20 µl of 1:1 mix of Magnetic bead suspension and Proteinase K solution (40 µg/µl). KingFisher Flex, loop though 3 times: 2 min fast mix, 30 s half mix, 30 s bottom mix
4. Employ magnetic separation (Collect beads, 3x10 s)
5. Wash beads with 400 µL VHB wash buffer (Release beads, 3 min fast mix, collect beads 3x5 s)
6. Wash beads with 500 µL SPR Wash buffer (Release beads, 2 min fast mix, collect beads 3x5 s)
7. Wash beads with 500 µL SPR Wash buffer (Release beads, 2 min fast mix, collect beads 3x5 s)
8. Air dry magnetic beads (10 min, above well)
9. Elute nucleic acids in 50 µl nuclease-free water (Release beads, 5 min medium mix, collect beads 3x10 s)

Protocol in brief (not tested with home-made solutions; based on Omega April 2020 protocol)

1. Per sample, mix 240 µl lysis buffer and 1 µl carrier RNA (1 µg/µl)
2. Add 200 µl of patient sample in VTM, mix thoroughly; incubate for >15 min
3. Add isopropanol bead mix (280 µl isopropanol and 2 µl magnetic beads). KingFisher Flex, loop though 3 times: 2 min fast mix, 30 s half mix, 30 s bottom mix
4. Employ magnetic separation (Collect beads, 3x10 s)
5. Wash beads with 350 µL VHB wash buffer (Release beads, 3 min fast mix, collect beads 3x5 s)
6. Wash beads with 350 µL SPR Wash buffer (Release beads, 2 min fast mix, collect beads 3x5 s)
7. Wash beads with 350 µL SPR Wash buffer (Release beads, 2 min fast mix, collect beads 3x5 s)
8. Air dry magnetic beads (10 min, above well)
9. Elute nucleic acids in 50 µl nuclease-free water (Release beads, 5 min medium mix, collect beads 3x10 s)

Reagents

Guanidine thiocyanate based

GnSCN Lysis Buffer (TNA Lysis buffer equivalent)

	Amount for 1 L	Concentration
Guanidine thiocyanate	473 g	4 M
Sarkosyl (sodium lauroyl sarcosinate)	20 g	2%
1 M Tris-HCl pH 7.5	50 ml	50 mM
0.5 M EDTA pH 8.0	20 ml	10 mM
β-mercaptoethanol	10 ml	1%

GnSCN Wash buffer 1 (VHB Wash buffer equivalent)

	Amount for 1 L	Concentration
Guanidine thiocyanate	118 g	1 M
1 M Sodium citrate pH 7*	10 ml	10 mM
Ethanol	560 ml	56%

*to set pH, add 10 ml 1 M HCl for every 500 ml 1 M Sodium citrate.

Guanidine hydrochloride based

GnHCl Lysis Buffer (TNA Lysis buffer equivalent)

	Amount for 1 L	Concentration
Guanidine hydrochloride	573 g	6 M
Sarkosyl (sodium lauroyl sarcosinate)	20 g	2%
1 M Tris-HCl pH 7.5	50 ml	50 mM
0.5 M EDTA pH 8.0	20 ml	10 mM
β-mercaptoethanol	10 ml	1%

GnHCl Wash Buffer 1 (VHB Wash buffer equivalent)

	Amount for 1 L	Concentration
Guanidine hydrochloride	107 g	1.25 M
1 M Sodium citrate pH 7*	10 ml	10 mM
Ethanol	560 ml	56%

*to set pH, add 10 ml 1 M HCl for every 500 ml 1 M Sodium citrate.

SPR Wash buffer 2 equivalent

80% Ethanol

Proteinase K (PCR grade; e.g. Roche Cat. No. 03115801001)

Dissolve in 10 mM Tris pH 8.0, 1 mM EDTA at 40 mg/ml

Store aliquots at -20°C

Carrier RNA (e.g. yeast tRNA, Roche Cat. No. 10109509001)

Dissolve in 10 mM Tris pH 8.0, 1 mM EDTA at 1 mg/ml

Store aliquots at -20°C

S2 Protocol References

1. Cervantes-Barragan L, Zust R, Maier R, Sierro S, Janda J, Levy F, et al. Dendritic cell-specific antigen delivery by coronavirus vaccine vectors induces long-lasting protective antiviral and antitumor immunity. mBio. 2010;1(4). Epub 2010/09/17. doi: 10.1128/mBio.00171-10. PubMed PMID: 20844609; PubMed Central PMCID: PMCPMC2939679.
2. Thiel V, Siddell SG. Reverse genetics of coronaviruses using vaccinia virus vectors. Curr Top Microbiol Immunol. 2005;287:199-227. Epub 2004/12/22. doi: 10.1007/3-540-26765-4_7. PubMed PMID: 15609513.
3. Burton JE, Easterbrook L, Pitman J, Anderson D, Roddy S, Bailey D, et al. The effect of a non-denaturing detergent and a guanidinium-based inactivation agent on the viability of Ebola virus in mock clinical serum samples. J Virol Methods. 2017;250:34-40. Epub 2017/09/25. doi: 10.1016/j.jviromet.2017.09.020. PubMed PMID: 28941617.
4. Pastorino B, Touret F, Gilles M, de Lamballerie X, Charrel RN. Heat Inactivation of Different Types of SARS-CoV-2 Samples: What Protocols for Biosafety, Molecular Detection and Serological Diagnostics? Viruses. 2020;12(7). Epub 2020/07/11. doi: 10.3390/v12070735. PubMed PMID: 32646015; PubMed Central PMCID: PMCPMC7412566.
