# Supplementary material for: A sensitive and affordable multiplex RT-qPCR assay for SARS-CoV-2 detection
Source: PLoS Biol. 2020 Dec 15;18(12):e3001030. doi: 10.1371/journal.pbio.3001030 (PMC7771873; doi:10.1371/journal.pbio.3001030)
Supplement: Attachment — Submitted filename: 109779_1_rebuttal_1902779_qwz7cq.docx [file pbio.3001030.s015.docx]

*We are grateful to the referees for investing valuable time in reviewing our work, and for recognising the importance and utility. We thank them for their insightful and constructive comments that have helped us significantly improve the manuscript.*

*Below, we provide a point-by-point response to all specific questions raised.*

Reviewer #1 (Evidence, reproducibility and clarity (Required)):

In order to improve SARS-CoV-2 diagnostics, Reijns et al. developed a multiplexed RT-qPCR protocol that allows simultaneous detection of two viral genes, one housekeeping gene as well as an external gene as an extraction control. Compared to running parallel assays to detect genes individually, the turnaround time is much shorter and reagents are saved. Furthermore, the presented data suggest that the assay is more sensitive than commercial kits. The authors also propose the detection of the human housekeeping gene as a measure of sample quality control. In principal, this work has potential but the manuscript itself needs a better structure.

**Major concerns:**

The authors have used the Takara RT-qPCR kit for their study. Did the authors try other commercial kits?

*We have not assessed other commercial kits as the Takara reagent performed well, and has been easy to source. We expect that other one-step kits could be used if the need arose.*

*When we initiated this work in March 2020, we selected the Takara One Step PrimeScript™ III RT-PCR Kit based on 1) the practical advantages of a one-step reaction mix, 2) published evidence of its successful use in SARS-CoV-2 detection (see below), 3) availability in sufficient quantities for testing at scale, and 4) affordability.*

*(Published evidence: One of the first descriptions of an assay to detect SARS-CoV-2 [1] employed the Takara One Step PrimeScript™ III RT-PCR Kit, and this kit was later shown by others to perform as well as or better than Qiagen Quantifast Multiplex RT-PCR +R mastermix, ThermoFisher TaqPath 1-Step RT-qPCR MasterMix and ThermoFisher Taqman Fast Virus 1-step mastermix, when used to detect SARS-CoV-2 RNA from nose and throat swabs with N1, N2 or N gene assays [2].)*

Can the authors elaborate on the supply chain of the Takara kit?

*We have not had problems securing the Takara kit in sufficient quantities and in a timely fashion, and did so through the company’s Scotland and NE England representative. The managing director of Takara Bio Europe provided the following statement, as a clarification of the supply chain:*

*“Takara Bio Inc. has worked on significantly increasing the production of one-step RT-qPCR reagents to cover worldwide needs for SARS-CoV-2 detection. The production of this kit is based in China under ISO13485 certification and the European stock is based in, and distributed, from Paris. Throughout this pandemic, Takara Bio Europe has supplied millions of reactions around Europe to COVID-19 testing labs, without encountering any shortages or significant shipping delays.”*

Could it cover population testing in case of shortages of other commercial kits?

*Yes, it could. The Takara kit is available in 4,000 and 20,000 reaction pack sizes and therefore could well be a useful option in case of shortages of other commercial kits. Indeed, one motivation for developing the multiplex assay was to ensure diagnostic testing resilience in the face of reagent shortages.*

For better comparison, is it possible to give information on which primers the commercial kits are based on?

*We contacted both ThermoFisher and Abbott to ask for more information on the primers and probes included in the TaqPath COVID‐19 Combo Kit (detects N, ORF1ab and S gene) and Abbott RealTime SARS‐CoV‐2 assay (detects RdRp and N gene). Unfortunately, we were informed that this information is proprietary. For clarity, we have included the following in the Materials and Methods section:*

*“Primers and probes included in the TaqPath COVID-19 Combo Kit (Thermo Fisher Scientific, Cat. No. A47814) detect SARS-CoV-2 ORF1ab, N and S gene; those in the Abbott RealTime SARS-CoV-2 assay (Cat. No. 09N77-090) detect RdRp and N gene. Further details are not available, as this information is proprietary.”*

Also, explain better the primers used in this study. For example, the N1 and N2 primers are directed against different regions of the SARS-CoV-2 N gene.

*We thank the reviewer for encouraging us to better explain the primers we use for our own assays, and now provide more detailed information in a new Fig 1.*

The result section needs a better structure as the first two pages do not refer to any of the main figures. For example, in which figure or table can the reader find the data that are discussed in lines 83 to 87?

*We have now substantially re-structured the entire Results section, and include the data that was discussed in lines 83 to 87 of the original manuscript, in Fig 1D of the revised manuscript.*

Table S1, instead of current Table 1, could be moved to main figures as it contains the important finding that the multiplexed assay may be more sensitive than the commercial one.

*As suggested, we have moved Table S1 to the main display items (now Table 1), and moved the original Table 1 to the supplementary items (now Table S3).*

The authors identified some samples that scored negative in commercial assays but positive in their new assay. This is important, however, the possibility of detecting false positives should be strengthened in a "Discussion" section.

*We thank the reviewer for highlighting this, and now discuss the issue of detecting false positives in more detail in the Discussion section of the revised manuscript:*

*“RT-qPCR tests are molecular tests with high intrinsic accuracy, however false positive and false negative results can occur. The use of multiplex assays that detect multiple SARS-CoV-2 targets, such as those reported here, reduces the chance of both. Off-target reactivity is one possible cause of false positives, and although some have reported high false positive rates for the E gene assay [20, 22], this does not match our experience. In two patients, our N1E-RP and N2E-RP assays detected virus, albeit weakly, whereas commercial assays did not. As multiple SARS-CoV-2 targets were positive, these are likely true positive results and not due to off-target reactivity. False positives can also occur due to lab issues such as sample mislabelling, data entry errors, reagent contamination with target nucleic acids or contamination of primary specimens. However high standards of quality control at all stages of testing, and effective mitigation strategies should quickly identify problems. Additionally, sample re-test with an independent assay and/or patient re-sampling should also be effective measures to counter false positives, particularly in low pre-test probability situations such as mass screening.”*

Figures 1 to 3 have different panels which seem to be redundant. For example, Fig 1 A and B, Fig 2 B and C, Fig 3 C and D.

*These panels did contain the same data, plotted to convey slightly different information. However, we agree that this introduces a level of redundancy. For enhanced clarity, in the revised manuscript, we have removed most of these panels altogether, or moved them to supplementary figures.*

Figure 1: Give a rational why comparing before and after extraction. This heavily depends on the extraction method and not on the detection itself. In addition, IVT RNA does not reflect the complexity of a clinical specimen. This is rather confusing and deviates from the important findings.

*As part of the validation procedure it was important for us to show that the entire workflow, including the extraction procedure, was robust for use in clinical diagnostics. In this context, comparing pre- and post-extraction RT-qPCR results for both IVT RNA and viral samples provided us with an opportunity to test extraction efficiency. However, we agree that for the purpose of this manuscript, the inclusion of these data in (the former) Fig 1 detracted from the main message. In the revised manuscript we have therefore moved the data comparing Cq values before and after extraction to a new Fig S1, and briefly state the rationale behind this in the main text and figure legend.*

*It was not our intention to imply that IVT RNA in any way reflects the complexity of a clinical specimen. We include these data as part of the step-by-step validation of our assays. Firstly, we show high sensitivity using IVT RNA; secondly, we show that a similar sensitivity is achieved on viral positive controls; and thirdly, we show that our assays perform equally well to widely used commercial assays on clinical samples.*

Figure 3: Were any of the negative samples/patients tested with an undetectable housekeeping gene, re-test positively?

*None of our patient samples had undetectable levels of RPP30. We note that all NTS samples were collected by healthcare professionals and in this context such findings will likely be rare. However this may not be the case when dealing with samples obtained by self-swabbing as the reviewer highlights in a comment below.*

Did adding this housekeeping gene as a control actually improve the detection of any patient samples? If the authors want to convince the readership of this quality control, experimental evidence should be provided.

Fig 3C and D seem to contain this information somewhat, as here, the values were normalized and the CT values for the E and N gene decreased. Nevertheless there is no real explanation of this figure provided in the Result section at all. While this figure has potential, the authors have to keep in mind that the number of cells in a swab can be affected by many biological factors, including age, sample timing, inflammation of the respiratory tract, etc. In addition, viral genomes can exist intra- as well as extracellular, in the form of free virus. So even in the absence of human cells/detectable housekeeping genomes, viral RNA can be or should be present in a sample in case of infection. This explains (probably) why a correlation between detectable housekeeping gene and viral RNA is absent (Fig 3A and B?). This entire Fig 3 just needs a better explanation. The provided text does not describe any results and should go into a "Discussion" section.

*We thank the reviewer for highlighting the need to explain Fig 3 more clearly and that a key question is whether there is a correlation between the levels of the housekeeping control and viral RNA. Prompted by this question, we reanalysed our data and now show that there is a strong and statistically significant positive correlation between Cq values for RPP30 and SARS-CoV-2 targets (see below, and new Fig 4C). This shows that there is a lower probability of detecting SARS-CoV-2 RNA in samples that contain fewer human cells. This likely implies that for samples with high RPP30 Cq values, a proportion of virus positive samples will be missed, contributing to the high false negative rates that have been reported [3-5].*

*Providing additional experimentation would require systematic re-contacting and re-testing of cases, and this is beyond our current research framework. While outside the scope of the current study, we hope that* *our manuscript will encourage others to perform the necessary large-scale experiments. Nonetheless, with this correlation alone, we believe that RPP30 provides useful information of benefit to clinical diagnostics (also see our response to Reviewer 2), and in the revised manuscript we outline how it might be best utilised (Discussion, Table S6).*

*To provide a better explanation of Figure 3 (now Fig 4), we have included the following in the Results section:*

*“A statistically significant linear correlation between Cq values for each of the viral probes (E, N1, and N2) and the Cq values for the RPP30 sample quality probe (p < 0.001; Fig 4C and data not shown) established that samples containing fewer human cells are more likely to have less SARS-CoV-2, decreasing the chance of detection. To visually demonstrate the impact of this, we normalised SARS-CoV-2 Cq values to the sample with the lowest amount of human material detected (reference sample, RPP30 Cq value 31.7/32.1). Due to the linear correlation between RPP30 and viral Cq values, subtracting the difference in RPP30 Cq between a particular positive sample and the reference sample from the SARS-CoV-2 Cq gives an indication of what the viral Cq may have been had it contained the same low amount of human material present in the reference sample. This shows that of the 26 positive samples we detected, between 4 and 6 (15-23%) would not have tested positive, with at least one of the viral targets exceeding the detection limit (Cq > 40; Fig 4D and Fig S4A). Theoretically, using this approach, even a strong positive sample (SARS-CoV-2 Cq value of 28.2) of good quality (RPP30 Cq value of 20.3) may have given a false negative test result (SARS-CoV-2 Cq value of 40) if it had contained the same low amount of human material as the reference sample (RPP30 Cq value of 32.1; viral Cq: 32.1-20.3+28.2=40). Conversely, normalising samples to an optimal quality sample (RPP30 Cq 20.1/20.3) gives an indication of what viral Cq values may have been if all samples had contained a similar (more optimal) amount of material (Fig 4E, Fig S4B). This highlights the possibility that a proportion of apparent SARS-CoV-2 negative samples are in fact false negatives as a result of insufficient material in the swab fluid.”*

Self-swabbing is surely a potential source of variability and false-negatives, but many publications have shown the suitability of saliva testing. This should also be discussed and would probably negate the need for such a quality control.

*We agree with the reviewer that self-swabbing will be more prone to variability. Therefore, the RPP30 control will have particular value here, lowering the associated risk of false negatives. While NTS sampling remains a major modality for testing for the foreseeable future, saliva is certainly a potential alternative strategy, one that may benefit from lower sample variability.*

*We now include the reviewer’s point on this in the Discussion:*

*“Testing saliva, as an alternative to NTS sampling, could also be beneficial as a modality that may have less-sample to sample variability [7]”*

Which assay works better, the N1E-RP or the N2E-RP assay? A final conclusion is missing here.

*Although we could not detect substantial differences between these two assays during our validation process, others have reported a marginally higher sensitivity of the N1 over the N2 assay [6]. We would therefore recommend the use of the N1E-RP assay for first line testing, with the N2E-RP assay available as a second line test of equivalent sensitivity in case of inconclusive initial test results. We comment on this in the revised manuscript:*

*“Although we did not detect substantial differences between our two assays, others have reported higher sensitivity of the N1 over the N2 assay [19]. We therefore recommend the use of the N1E-RP assay for primary testing, and the N2E-RP assay could be employed if initial results are inconclusive.”*

Reviewer #1 (Significance (Required)):

Naturally, in this pandemic, this topic is important as sensitive and affordable methods to detect SARS-CoV-2 infections are in need. This Reviewer agrees that multiplexing could be an elegant approach to fill this need.

Reviewer #2 (Evidence, reproducibility and clarity (Required)):

In this manuscript, Reijns and colleagues describe an approach to detect the causative agent of COVID-19, the beta coronavirus SARS-CoV-2, using an inexpensive in-house multiplex RT-qPCR. Concomitantly, viral E, N and RdRP(probe P2) as well as human RPP30 and a herpesvirus nucleic acid are also detected in order to monitor both the sample quality and the sample preparation.
Reijns et al. performed testing on a huge amount of samples and used the data to describe the strength and limitations of the assay. The data is sound and give a very good impression of the 4-plex PCR capabilities. I read manuscript fluently and consider as linguistically very good.
However, I still have a few comments and remarks that would strengthen the manuscript:

**Major issues:**

In the first section of the results section, many primer / probe conditions are given that make the reading flow difficult. Instead of using (data not shown) it would be helpful to use a table or a graphic to illustrate the various approaches.

*We thank the reviewer for suggesting the use of graphics to explain our different approaches. To aid the reader, we now include a diagram in the new Fig 1 that shows the positions of primers and probes used in our work (A), and illustrate the various 4-plex assays (B, C).*

In general, I suggest to replace Ct by Cq, since the IVT standards are a quantification method.

*As suggested by the reviewer we now use Cq instead of Ct throughout our manuscript, following MIQE guidelines [7].*

There has already been a change away from the initial E and RdRP gene based assay because of the published sensitivity issues and the use of degenerate bases as well as the detection of unspecific nucleic acids for E gene). In particular, it has been shown that the Sarbeco-E-yields false positive results (Toptan et al. 2020 (<https://doi.org/10.3390/ijms21124396>), Konrad et al. 2020 (<https://doi.org/10.2807/1560-7917.ES.2020.25.9.2000173>)), so that many laboratories do not consider E-gene-based results for borderline samples anymore. In this manuscript, the authors should comment on why they still use the results from the E gene / RdRP and describe their experience.

*We thank the reviewer for highlighting issues with the RdRp and E gene primer/probe pairs. In the process of our work we had also become aware that the RdRp-P2 assay suffers from low sensitivity, as has now been widely reported. However, although the E gene assay also detects SARS-CoV, we were not aware of potential problems with high rates of false positives as described by Toptan et al (2020) and Konrad et al (2020). It did come to our attention that early on in the pandemic some oligonucleotide producers reported problems with contamination of primers with SARS-CoV-2 template RNA synthesised in the same facilities, and were careful to avoid these providers. In our work, we did not experience any problems with apparent false positive detection of the E gene: it was never detected in any of our negative controls, and out of 84 patients that tested negative with the commercial TaqPath assay we did not find any that were positive for E gene only when using our N1E-RP and N2E-RP assays. In this context, it is also important to emphasise that a positive diagnosis is given only when both viral targets are detected (Table S6), which is one of the strengths of our multiplex assays.*

*As suggested by Reviewer 1, we now discuss the issue of false positives in more detail in the revised manuscript. We also comment on high false positive rates observed by others for the E gene assay, citing the two studies, but state that this does not match our own experience:*

*“Off-target reactivity is one possible cause of false positives, and although some have reported high false positive rates for the E gene assay [20, 23], this does not match our experience.”*

In this manuscript, it should be indicated that the SARS-CoV-2 specific Probe P2 (according to Corman et al. 2020) was used. The reason for lower sensitivity due to nucleotide ambiguity and mismatch has to be explained in more detail. In addition to Corman et al. 2020 (see reference 2), Toptan et al 2020 (<https://doi.org/10.3390/ijms21124396>) might serve as helpful literature.

*In tables describing primers and probes, and the new Fig 1, we indicate that we used RdRp probe P2. In addition, we now also specifically state in the legend of Fig 1 that this probe only detects SARS-CoV-2 and that the primers used in the RdRp-P2 assay (as originally designed by Corman et al) contain nucleotide ambiguities and a mismatch. Finally, in the main text we explain:*

*“Overall, we find RdRp detection to be at least 20-fold less sensitive than for E gene, N1 and N2 under our assay conditions; consistent with reports by others [19]. This may be due to a mismatch in the reverse primer employed in the RdRp (P2) assay, as originally designed [14].”*

With regard to the marginally positive samples that were not consistent in all assays, were the PCR products analyzed using high-resolution PAA genes and, if possible, sequenced? The sequencing approach (Sanger or NGS) offers the final characterization of the PCR products (especially for pan-genotypic primers such as E-Sarbeco). The samples declared as "inconclusive" could be further characterized in this way.

*Unfortunately, it has not been possible for us to carry out additional analyses for such (now historical) samples. Given the high prevalence of SARS-CoV-2 and the low sequence variability at primer/probe binding sites (new Table 2 and S5), inconclusive or marginally positive samples most likely reflect low viral load and/or low sample quality. Nevertheless, we now highlight the utility of further characterising such samples in the revised manuscript:*

*“However, differentiating between samples with low viral loads and false positives is challenging. Analysis of such samples by Sanger sequencing of PCR products, or nanopore sequencing of RNA present could provide useful information. Further clinical evaluation and repeat sampling of the patient involved may also be a beneficial route to a secure clinical diagnosis.”*

The normalization in figure 3 should be also explained in the main text. Especially, why this approach was used for normalization.

*In the Results section we now describe the normalisation as follows:*

*“A statistically significant linear correlation between Cq values for each of the viral probes (E, N1, and N2) and the Cq values for the RPP30 sample quality probe (p < 0.001; Fig 4C and data not shown) established that samples containing fewer human cells are more likely to have less SARS-CoV-2, decreasing the chance of detection. To visually demonstrate the impact of this, we normalised SARS-CoV-2 Cq values to the sample with the lowest amount of human material detected (reference sample, RPP30 Cq value 31.7/32.1). Due to the linear correlation between RPP30 and viral Cq values, subtracting the difference in RPP30 Cq between a particular positive sample and the reference sample from the SARS-CoV-2 Cq gives an indication of what the viral Cq may have been had it contained the same low amount of human material present in the reference sample. This shows that of the 26 positive samples we detected, between 4 and 6 (15-23%) would not have tested positive, with at least one of the viral targets exceeding the detection limit (Cq > 40; Fig 4D and Fig S4A). Theoretically, using this approach, even a strong positive sample (SARS-CoV-2 Cq value of 28.2) of good quality (RPP30 Cq value of 20.3) may have given a false negative test result (SARS-CoV-2 Cq value of 40) if it had contained the same low amount of human material as the reference sample (RPP30 Cq value of 32.1; viral Cq: 32.1-20.3+28.2=40). Conversely, normalising samples to an optimal quality sample (RPP30 Cq 20.1/20.3) gives an indication of what viral Cq values may have been if all samples had contained a similar (more optimal) amount of material (Fig 4E, Fig S4B). This highlights the possibility that a proportion of apparent SARS-CoV-2 negative samples are in fact false negatives as a result of insufficient material in the swab fluid.”*

Nonetheless, it looks like the normalized values wills cluster much more strongly than those corresponding to the actual values. The authors should comment on this phenomenon. It appears that the higher cq values (less virus) are subject to a strong correction factor more often than high values. Are there any statistical relevant tendencies towards this phenomenon? For everyday clinical practice, does this mean that low samples Cqs (mostly) only reflect the quality of the sample, but not the viral load?

*We thank the reviewer for highlighting the stronger clustering of Cq values after normalisation, and for encouraging us to explore this further. We now show that there is a statistically significant linear correlation between RPP30 and SARS-CoV-2 Cq values (Fig 4C). This would indeed imply that a substantial proportion of the variability in SARS-CoV-2 Cq values seen in clinical practice is due to sample quality rather than different viral loads. However, outliers from the linear correlation when comparing samples from many different patients are to be expected (as seen in Fig 4C), because viral load is known to vary, with time of sampling relative to onset of symptoms one important contributing factor. In a research context, expressing viral load relative to a human control may be beneficial to differentiate between sample quality and absolute quantities of (intra/extracellular) viral RNA.*

*In the revised manuscript we state:*

*“Notably, the SARS-CoV-2 Cq values clustered more strongly after normalisation (Fig 4D, E; Fig S4). This reduced variability not only shows that the amount of human material present in NTS samples impacts on assay sensitivity, but also suggests that variability in viral load is not as great as implied by RT-qPCR data without normalisation.”*

Finally, it remains somewhat unclear to what extent the Cq values of the RPP30 should have an influence on the routine diagnostics. The authors discuss that a fixed cutoff value would be a possibility to sort out poor swab samples, but if a cq value is available it would also make sense to generate a kind of quality score that can display the significance of a test. It would be helpful if the authors could comment on this or other possibilities.

*We agree that it would be beneficial for routine diagnostics to derive such a measure. However, at this stage we do not have sufficient data to generate a robust quality score based on the RPP30 Cq values. Nonetheless, we believe RPP30 Cq values have immediate utility for routine diagnostics, and could help improve validity of test results going forward:*

*1. Samples with undetectable RPP30 should trigger repeat sample collection, and not be given a false negative test result;*

*2. Samples with high viral Cq values and/or for which only one of two viral targets are detected can be better interpreted in the context of the amount of human material as measured by RPP30 Cq;*

*3. Ongoing monitoring of swab quality allows rapid identification of potential technical issues with swabbing;*

*4. Normalisation of viral Cq values using RPP30 Cq values might be helpful in a research context to derive a more meaningful measure of viral loads, by removing one source of variability;*

*5. Collection of such data on an ongoing basis would ultimately allow this to be translated into a quality score that could be used as part of diagnostics algorithms.*

*In the revised manuscript we now discuss this as follows:*

*“Absence of RPP30 signal (undetected or Cq >40) clearly indicates that absence of viral detection cannot be interpreted as a negative test result and that a repeat test is required (Table S6). However, utilising RPP30 Cq values when interpreting an apparent SARS-CoV-2 negative sample requires further consideration: what should the RPP30 Cq limit be for which to order a repeat test? One option would be to simply set an arbitrary cut-off, e.g. one could decide to re-test any samples with RPP30 Cq >30, or with Cq values above the 95th centile (Cq ~ 31 for our 108 samples). To determine robust cut-off limits, collection of RPP30 data for a much larger number of patient samples would be desirable. This would allow development of diagnostic algorithms that could incorporate a sample quality score based on the level of RPP30 detected. Nonetheless, RPP30 data, even as it stands, are useful for the interpretation of cases for which only one of the SARS-CoV-2 targets is (weakly) positive, with samples with high RPP30 Cq values interpreted with particular caution. In such cases, repeat testing of the same sample (with an independent assay of equal or better sensitivity) would be advisable, and repeat patient specimen collection and testing might also be considered (see Table S6 for guidance).”*

Over the past few months, more and more virus subtypes have formed through the manifestation of point mutations (and amino acid substitutions). The authors should therefore definitely comment on the current strains as to whether all primers / probes are able to detect the virus variants circulating worldwide without loss of sensitivity.

*We thank the reviewer for this suggestion and now include a table providing information on mismatches in primer and probe binding sites (see Table 2 and Table S5 of the revised manuscript). This shows that only a small proportion of 97,782 strains for which high quality genome sequencing is available have changes in primer/probe binding sites. In addition, the use of two different primer/probe sets in our multiplex assays provides a further safeguard against failure to detect strains with such changes.*

Along this line, which virus strains were used for the cultivation as described in line 131? Is sequence data available? If so, it would provide helpful information to characterize the viral strain.

*We have added strain information, accession codes for genome sequences and information on primer/probe binding for both control strains (hCoV-19/England/02/2020 and BetaCoV/Munich/ChVir984/2020) we used in our work (see Materials and Methods of the revised manuscript).*

Line 206ff: In my opinion, this section belongs more to the discussion part than to material and methods that describe the technical implementation.

*We agree and have now moved this section to the Discussion. Furthermore, we’ve made additional changes to better highlight the potential for further improvements to our assays, and SARS-CoV-2 RT-qPCR assays in general.*

Is there a loss of sensitivity compared to the single PCRs? This data is very important and useful for other users. They should therefore be included explicitly in the manuscript (supplements).

*We set out to develop multiplex PCR assays to allow more efficient and cost-effective testing. In the early stages of this process we performed small pilot experiments with positive control IVT RNA and individual primer/probe pairs that are widely used and well-established to sensitively detect SARS-CoV-2 RNA. With the exception of the RdRp primers/probe, we found all to perform well, with the ability to detect 10 copies of RNA. However, we did not perform a side-by-side comparison of uni- and multiplex PCRs, and to improve the structure and flow of the Results section, as requested by Reviewer 1, we have now removed all mention of the single PCR assays.*

*Altogether, the key message of our work is that the N1E-RP and N2E-RP assays are able to detect between 1 and 3 copies of SARS-CoV-2 RNA and show equivalent performance to commercially available multiplex assays.*

**Minor issues:**

Line 15 ff.: Source is missing, is this WHO-data?

*The estimated number of infections and fatalities at the time of writing of the original manuscript was based on data from the online interactive dashboard hosted by Johns Hopkins University. At the suggestion of Reviewer 3, we have removed precise numbers from the revised manuscript to make the introduction less time-dependent. Nonetheless, we now include a reference to the JHU online resource, as well as the weekly epidemiological updates from the WHO (*[*https://www.who.int/emergencies/diseases/novel-coronavirus-2019/situation-reports*](https://www.who.int/emergencies/diseases/novel-coronavirus-2019/situation-reports)*) for readers interested in the latest figures.*

Fig S3: How was the digital droplet PCR carried out? A brief description should be included in the legend text.

*We purchased these samples from QCMD, an independent International External Quality Assessment & Proficiency Testing (EQA/PT) organisation. QCMD performed the digital droplet PCR, before distribution under the QCMD 2020 Coronavirus Outbreak Preparedness EQA Pilot Scheme, and provided us with details, which have now been added to the Materials and Methods section:*

*“Quantification of control samples was carried out by QCMD prior to distribution within the EQA scheme, using droplet digital PCR (ddPCR) with E-gene primers and probe [13, 14] on the Biorad droplet digital PCR platform. A serial dilution of inactivated SARS-CoV-2 (strain BetaCoV/Munich/ChVir984/2020; GenBank Accession MT270112, [32]) was prepared and each dilution replicate tested 4 times using both RT-qPCR and ddPCR assays. Regression analysis was used to assess the linearity across the dilution series, and the analytical measurement range established for both assays, comparing results of each by Bland-Altman difference plot."*

*In addition, we provide more details with the relevant table (new Table S3) and in the legend of the associated figure (new Fig 2) we state: “See Materials and Methods for details”.*

Figure 1a: PCR efficiencies are missing.

*We have now added PCR efficiencies to all relevant graphs.*

Line 145: MS2 appears, but without explaining the context. This should be improved here with additional information (this does not appear until line 154).

*At first mention of MS2 in the main text, we now state:*

*“Internal controls were included to provide confirmation of successful nucleic acid extraction and absence of PCR inhibitors, with lysis buffer spiked with both MS2 (an RNA bacteriophage that infects Escherichia coli) and PhHV (a DNA virus that infects seals), detected by the TaqPath and N1E-RP/N2E-RP assays respectively..”*

Page 15, H20 instead of H20, reaction mix instead of Reaction mix.

*In the supplementary protocol, we have changed “H2O” to “H_2_O” and “Reaction mix” to “reaction mix”.*

Reviewer #2 (Significance (Required)):

The novel coronavirus SARS-CoV-2 is the causative agent of the acute respiratory disease COVID-19 which has become a global concern due to its rapid spread and high death rate. While some patients have no symptoms at all, but are still able to spread the virus, others have severe symptoms, often with fatal outcome. The gold standard in SARS-CoV-2 detection is the RT-qPCR approach, however, the high cost commercial kits are available in limited amounts only.
The issue of the scarcity of resources is still an highly important issue, especially in terms of the incredibly rapidly increasing number of cases worldwide. Thus, the manuscript is of significance for the field and timely.
Especially, diagnostic laboratories in low-income countries that are involved in the managing the pandemic but also researchers will benefit from this manuscript and save resources.

Reviewer #3 (Evidence, reproducibility and clarity (Required)):

In this study Reijns et al developed a multiplex RT-PCR assay (alternative primer-probe sets and enzyme mixes) to detect SARS‐CoV‐2 and internal controls. The authors conclude that their assay performed equally well as established commercial kits. The authors also demonstrated that nose‐and‐throat swab samples have considerable variability in patient material content (>1,000‐fold variability). High variability is expected, but it is still important to substantiate this notion with numbers. Overall, I like the study and find it methodologically sound. Sample numbers in the tests are in most cases good. I have very few objections and hope to see the manuscript published soon.

**SPECIFIC COMMENTS:**

1."The COVID‐19 pandemic originated in Wuhan (China) in December 2019 and at the time of writing has infected more than 13.1 million people worldwide, resulting in well over 0.57 million COVID‐19‐related deaths..." I suggest a more timeless starting of the introduction, not pointing out exact number of infections and deaths since these numbers quickly become obsolete. The reader will know the severity of the pandemic and the importance of methodological development without statement of exact numbers. This comment reflects my personal opinion and it is completely up to the authors to choose how to phrase this section.

*We agree with the reviewer that a more timeless start to the introduction makes more sense. Therefore, as suggested, we have changed this section of the manuscript, which now reads as follows:*

*“The COVID-19 pandemic, caused by the novel coronavirus SARS-CoV-2 [1], originated in Wuhan (China) in December 2019 and rapidly spread across the globe, resulting in substantial mortality [2, 3] and widespread economic damage. Until a vaccine becomes available, public health strategies centred on reducing the rate of transmission are crucial to mitigating the epidemic, for which effective and affordable testing strategies to enable widespread population surveillance are essential.”*

2.Tables listing primers and probes should include the amplicon (PCR product) length for each primer-probe pair. Product length is an important consideration for fragmented RNA samples, such as for example heat-inactivated or longer-term stored samples. It should not be put on the reader to find out the amplicon lengths.

*To provide the reader with this information, the revised manuscript now includes the following:*

*1. As suggested, the amplicon length for each primer pair is added to all tables that list primers and probes (PCR products: RdRp – 100 bp; E- 113 bp; N1 – 72 bp; N2 – 67 bp);*

*2. A diagram in a new Fig 1 indicates the positions of all primers and probes on the SARS-CoV-2 genome along with amplicon length.*

*3. A supplementary SnapGene file with primers and probes on the SARS-CoV-2 (Wuhan-Hu-1) genome to allow readers to look at further details in the context of the viral genome.*

3.Line 131: "To confirm sensitivity using total viral RNA, nucleic acids isolated from cultured SARS‐CoV‐2 were also used to make a dilution series (10^‐1 to 10^‐6)." I lack a methodological description how viral nucleic acid was quantified. It is not entirely trivial to separate viral RNA from RNA contributed from the cells used for the in vitro expansion of the virus.

*We apologise for the lack of clarity on this in our original manuscript. The purpose of this experiment was not to measure a defined number of RNA molecules, but to ensure that there was no inhibition of viral target amplification in a more complex sample by demonstrating linearity over a range of dilutions. The cultured SARS-CoV-2 positive control was provided by Prof Rory Gunson (Clinical Lead West of Scotland Specialist Virology Centre, NHS Greater Glasgow and Clyde) as inactivated supernatant from virus (strain hCoV-19/England/02/2020) propagated in cell culture. We then isolated RNA from a dilution series of this supernatant, using the methods described in our manuscript, but did not determine the precise concentration. The RT-qPCR data for this series shows a good fit and amplification efficiency, similar to what was found for the IVT RNA, and QCMD virus calibration curves (new Fig 2). The known copy number of the QCMD virus (as determined by ddPCR) allowed us to calculate that the concentration of the virus in the supernatant provided to us was between 0.7 and 2.2 x 10^5^ copies/ml, with viral RNA detected down to between 0.7 and 3 copies with our N1E-RP and N2E-RP assays. We have substantially restructured the results section, and hope to have made the way we used the different viral controls clearer in the revised version of the manuscript.*

4.Line 150: "All positive and negative controls gave the expected results (Table S4)" I don't like the exact formulation since it is not clear for the reader what are the "expected results", including the "expected" quantitative results (Ct).

*We agree that the use of “expected results” does not provide the reader with sufficient information. We have therefore changed this to:*

*“Results for controls were as anticipated (Table S4), with signal absent (undetermined) for SARS-CoV-2 and RPP30 targets for the negative controls, and Cq values for the SARS-CoV-2 RNA positive control (50 copies) similar to those obtained previously (Fig 2A).”*

*In addition, we now provide more information on the precise nature of the negative and positive controls with Table S4:*

*“-ve (extr), negative control with viral transport medium after RNA isolation (does not contain SARS-CoV-2 or human material; does contain PhHV);*

*-ve, negative control containing water only (should not contain any RNA)*

*+ve, positive control with in vitro transcribed RNA (50 copies; contains SARS-CoV-2 target RNA, does not contain human or PhHV nucleic acids)”*

Reviewer #3 (Significance (Required)):

This study provides an alternative multiplex RT-PCR assay to detect SARS-CoV-2 infection. I find the results important and useful for the research and medical community.

***Rebuttal references***

*1.	Zhu N, Zhang D, Wang W, Li X, Yang B, Song J, et al. A Novel Coronavirus from Patients with Pneumonia in China, 2019. N Engl J Med. 2020;382(8):727-33. Epub 2020/01/25. doi: 10.1056/NEJMoa2001017. PubMed PMID: 31978945; PubMed Central PMCID: PMCPMC7092803.*

*2.	Brown JR, O’Sullivan D, Pereira RP, Whale AS, Busby E, Huggett J, et al. Comparison of SARS-CoV2 N gene real-time RT-PCR targets and commercially available mastermixes. 2020:2020.04.17.047118. doi: 10.1101/2020.04.17.047118 %J bioRxiv.*

*3.	Arevalo-Rodriguez I, Buitrago-Garcia D, Simancas-Racines D, Zambrano-Achig P, del Campo R, Ciapponi A, et al. False-negative results of initial RT-PCR assays for COVID-19: A systematic review. 2020:2020.04.16.20066787. doi: 10.1101/2020.04.16.20066787 %J medRxiv.*

*4.	Watson J, Whiting PF, Brush JE. Interpreting a covid-19 test result. BMJ. 2020;369:m1808. Epub 2020/05/14. doi: 10.1136/bmj.m1808. PubMed PMID: 32398230.*

*5.	Woloshin S, Patel N, Kesselheim AS. False Negative Tests for SARS-CoV-2 Infection - Challenges and Implications. N Engl J Med. 2020;383(6):e38. Epub 2020/06/06. doi: 10.1056/NEJMp2015897. PubMed PMID: 32502334.*

*6.	Vogels CBF, Brito AF, Wyllie AL, Fauver JR, Ott IM, Kalinich CC, et al. Analytical sensitivity and efficiency comparisons of SARS-CoV-2 RT-qPCR primer-probe sets. Nat Microbiol. 2020. Epub 2020/07/12. doi: 10.1038/s41564-020-0761-6. PubMed PMID: 32651556.*

*7.	Bustin SA, Benes V, Garson JA, Hellemans J, Huggett J, Kubista M, et al. The MIQE guidelines: minimum information for publication of quantitative real-time PCR experiments. Clin Chem. 2009;55(4):611-22. Epub 2009/02/28. doi: 10.1373/clinchem.2008.112797. PubMed PMID: 19246619.*
